# Age-related alterations in metabolome and microbiome provide insights in dietary transition in giant pandas

**DOI:** 10.1128/msystems.00252-23

**Published:** 2023-06-05

**Authors:** Fangyuan Liu, Rengui Li, Yi Zhong, Xu Liu, Wenwen Deng, Xiaoyu Huang, Megan Price, Jing Li

**Affiliations:** 1 Key Laboratory of Bio-resources and Eco-environment (Ministry of Education), College of Life Sciences, Sichuan University, Chengdu, Sichuan, China; 2 China Conservation and Research Center for the Giant Panda, Dujiangyan, Sichuan, China; 3 Key Laboratory of State Forestry and Grassland Administration on Conservation Biology for Rare Animals of the Giant Panda State Park, Dujiangyan, Sichuan, China; 4 China Wildlife Conservation Association, Beijing, China; Institute for Systems Biology, Seattle, Washington, USA

**Keywords:** giant pandas, metabolomics, 16S rRNA, metagenomics, age, bile acid-gut microbiota axis

## Abstract

We conducted UPLC-MS-based metabolomics, 16S rRNA, and metagenome sequencing on the fecal samples of 44 captive giant pandas (*Ailuropoda melanoleuca*) from four age groups (i.e., Cub, Young, Adult, and Old) to comprehensively understand age-related changes in the metabolism and gut microbiota of giant pandas. We characterized the metabolite profiles of giant pandas based on 1,376 identified metabolites, with 152 significantly differential metabolites (SDMs) found across the age groups. We found that the metabolites and the composition/function of the gut microbiota changed in response to the transition from a milk-dominant diet in panda cubs to a bamboo-specific diet in young and adult pandas. Lipid metabolites such as choline and hippuric acid were enriched in the Cub group, and many plant secondary metabolites were significantly higher in the Young and Adult groups, while oxidative stress and inflammatory related metabolites were only found in the Old group. However, there was a decrease in the α-diversity of gut microbiota in adult and old pandas, who exclusively consume bamboo. The abundance of bacteria related to the digestion of cellulose-rich food, such as Firmicutes, *Streptococcus,* and *Clostridium,* significantly increased from the Cub to the Adult group, while the abundance of beneficial bacteria such as *Faecalibacterium*, *Sarcina,* and *Blautia* significantly decreased. Notably, several potential pathogenic bacteria had relatively high abundances, especially in the Young group. Metagenomic analysis identified 277 CAZyme genes including cellulose degrading genes, and seven of the CAZymes had abundances that significantly differed between age groups. We also identified 237 antibiotic resistance genes (ARGs) whose number and diversity increased with age. We also found a significant positive correlation between the abundance of bile acids and gut bacteria, especially *Lactobacillus* and *Bifidobacterium*. Our results from metabolome, 16S rRNA, and metagenome data highlight the important role of the gut microbiota-bile acid axis in the regulation of age-related metabolism and provide new insights into the lipid metabolism of giant pandas.

**IMPORTANCE:**

The giant panda is a member of the order Carnivora but is entirely herbivorous. The giant panda’s specialized diet and related metabolic mechanisms have not been fully understood. It is therefore crucial to investigate the dynamic changes in metabolites as giant pandas grow and physiologically adapt to their herbivorous diet. This study conducted UPLC-MS-based metabolomics 16S rRNA, and metagenome sequencing on the fecal samples of captive giant pandas from four age groups. We found that metabolites and the composition/function of gut microbiota changed in response to the transition from a milk-dominant diet in cubs to a bamboo-specific diet in young and adult pandas. The metabolome, 16S rRNA, and metagenome results highlight that the gut microbiota-bile acid axis has an important role in the regulation of age-related metabolism, and our study provides new insights into the lipid metabolism of giant pandas.

## INTRODUCTION

The giant panda is a member of the order Carnivora but is entirely herbivorous after weaning, feeding almost exclusively on bamboo. However, it retains a short carnivoran alimentary tract and, consequently, has very low digestive efficiency for cellulose-rich food. Unsurprisingly, the specialized diet and related metabolic characteristics in the giant panda have been of particular interest to researchers. The herbivorous giant panda has a metabolic rate below a mammalian carnivore of the same size but is similar to other herbivores (e.g., sloths) (1). Nie et al. argued that giant pandas maintain a low metabolic level through reductions in their activity levels, a shrinkage of major energy consuming organs, and a decline in thyroid hormone levels (2). Despite numerous studies on giant panda genomics (3), transcriptomics (4, 5), and gut microbiome composition (6), it is unclear how pandas digest bamboo with a low metabolic rate. It is also uncertain how metabolic characteristics change with increasing age, particularly during the transitionary period from a milk-based diet in cubs to a bamboo-based diet in young pandas.

Metabolomics technology is a powerful tool that can accurately and directly reflect the metabolic phenotypic changes of cells, tissues, and organisms in response to disease, aging, and environmental factors. (7). Metabolomics allows detection and identification of many metabolites at the same time using gas chromatography-mass spectrometry (GC-MS), liquid chromatography (LC)-MS, or nuclear magnetic resonance (NMR) (8). Metabolomics has been widely used to identify potential biomarkers associated with aging and diseases (9, 10). Previous metabolomics studies found that lipid metabolism and carbohydrate metabolism changed significantly with age in humans and animals. For example, the content of lipid metabolites such as lysophosphatidylcholine and sn-glycerol-3-phosphate choline decreased with advanced age in humans and could cause apoptosis of cells (11). Tricarboxylic acid cycle-related metabolites (i.e., carbohydrate metabolism), such as NAD+, decreased with increasing age, leading to an increase in the oxidative stress response and inflammatory reactions in elderly people (12). Metabolomics data also can be integrated with other omics data, such as genome, transcriptome, or microbiome, to address both physiological and pathological metabolic mechanisms. For instance, accumulating evidence suggests that there are direct and indirect relationships between the gut microbiome and fecal metabolites. Variations in the gut’s microbiota composition can induce metabolic shifts that may result in alterations of a host phenotype (13). In particular, recent studies have SDM demonstrated the involvement of the gut microbiota-bile acid axis in age-related metabolism (14, 15). Given the importance of the gut microbiota-bile acid axis in metabolism, several studies suggested that it was highly possible that the gut microbiota-bile acid axis was the underlying source of many age-related metabolic diseases, such as obesity, type 2 diabetes mellitus, non-alcoholic fatty liver disease, and atherosclerosis (16, 17).

There are few giant panda metabolomic studies, and these have mainly focused on seasonal changes in metabolites, metabolites after consumption of different bamboo parts, and the differences in metabolites between wild and captive pandas (18 - 20). Zhu et al. conducted metabolomic studies using^1^H NMR of giant panda feces, urine, serum, and saliva, and found that fucose may play an important role in the age-dependent metabolism changes of pandas (21). Yang et al. performed metabolome studies on giant panda serum from three age groups (young, adult, and old) and observed that fatty acid and lipid metabolism pathways were significantly altered with age (22). Although age-related changes in the giant pandas’ gut microbiota have been investigated using 16S rRNA (23), the relationship between the gut microbiota and metabolites, and how it changes in response to dietary transitions during the giant panda lifespan is unclear. This information will be essential to better understand the influences of the microbial community on metabolic function in giant pandas and subsequently wild and captive panda health.

We combined UPLC-MS-based metabolomics with 16S rRNA gene sequencing and metagenomics to comprehensively explore the dynamic changes in gut microbiota and fecal metabolites in captive giant pandas at different age stages. Fecal samples from four age stages: Cub, Young, Adult, and Old were collected from individuals at the Giant Panda Breeding Research Center (Dujuangyan, China). According to Center records, cubs are fed with breast milk and/or formula, young pandas are weaned onto a bamboo-dominant diet, and adult and old pandas feed almost exclusively on bamboo. Our combined results from multi-omics aimed to address (i) which metabolites and how metabolites changed with the dietary transition from milk in cubs to bamboo in young, adult, and old pandas; (ii) how the gut microbiome changed across the four age stages and supported the host’s nutrient acquisition from high cellulose bamboo; (iii) how lipid metabolism changed with age since the fat content is extremely low in the giant panda diet; and (iv) how does the gut microbiota-bile acid axis influence age-related metabolism changes in giant pandas given the important role of the gut microbiota-bile acid axis in human lipid metabolism (24). Addressing these issues will further our understanding of the giant pandas’ adaptation to the specialized, herbivorous diet and corresponding metabolic changes.

## MATERIALS AND METHODS

### Sample collection

Fresh fecal samples were collected from giant pandas living at the Giant Panda Breeding Research Base in Chengdu, Sichuan Province, People’s Republic of China. Fecal samples were collected from 44 captive giant pandas divided by age: Cub (1.1, 2 yr, *n* = 10), Young (3, 7 yr, *n* = 10), Adult (11, 18 yr, *n* = 12), and Old (20, 28 yr, *n* = 12). Individual and diet information is shown in Table S1. Samples were collected from September to November 2020. The fecal samples were collected in sterile 15 mL centrifuge tubes and labeled with the animal identification number and date of collection. All samples were stored at −80℃ for further metabolomics profiling and microbial community analysis. Each sample was then divided into three portions for metabolomics, 16S, and metagenomics.

### Metabolomics profiling

#### Sample processing

The fecal samples were slowly thawed at 4℃, and a proportion of the samples were added into a cold methanol/acetonitrile/water solution (2:2: 1, v/v). After eddy mixing, low temperature ultrasound for 30 min and being frozen at −20℃ for 10 min, the mixture was centrifuged at 14,000 g 4℃ for 20 min. The supernatant was dried in a vacuum centrifuge. During mass spectrometry analysis, 100 μL acetonitrile/water (1:1, v/v) was added to redissolve, then centrifuged at 14,000 g at 4℃ for 15 min, and then the supernatant was sampled for analysis. All samples were mixed to prepare QC samples.

#### UPLC-MS analysis conditions

Analyses were performed using an UPLC (1290 Infinity LC, Agilent Technologies) coupled to a quadrupole time-of-flight (AB SciexTripleTOF 6600). A ACQUIY UPLC BEH (2.1 mm × 100 mm, 1.7 µm) column (waters, Ireland) was used for HILIC separation of samples. In both ESI positive and negative modes, the mobile phase contained A=25 mM ammonium acetate and 25 mM ammonium hydroxide in water and B= acetonitrile.The gradient elution procedure was run under the following gradient parameters: 0–0.5 min, 95%B; 0.5–7 min, 95% – 65%B; 7–8 min, 65%–40%B; 8–9 min, 40%B; 9–9.1 min, 40–-95%B; and 9.1–12 min, 95%B. The samples were placed in an automatic sampler at 4℃ throughout the analysis.The ESI source parameters were set as curtain gas (CUR) as 30, source temperature: 600℃, Ion Source Gas1 (Gas1) as 60, Ion Source Gas2 (Gas2) as 60, and IonSpray Voltage Floating (ISVF) ± 5500 V. In MS only acquisition, the instrument was set to acquire over the *m*/z range 60–1000 Da, and the accumulation time for TOF MS scan was set at 0.20 s/spectra. In auto MS/MS acquisition, the instrument was set to acquire over the *m*/z range 25–1000 Da, and the accumulation time for product ion scan was set at 0.05 s/spectra. The product ion scan was acquired using information dependent acquisition (IDA) with high sensitivity mode selected. The parameters were set as: decluttering potential (DP), 60 V (+) and −60 V (−); the collision energy (CE) was fixed at 35 V with ± 15 eV; exclude isotopes within 4 Da, candidate ions to monitor per cycle: 10. To avoid the influence caused by the fluctuation of the instrument detection signal, QC samples were inserted into the sample queue to evaluate the stability of the system and the reliability of the experimental data.

#### Metabolomics data processing

The original data in WIFF format were converted into mzXML format by Proteowizard, and then XCMS software (25) was used for alignment of peak intensities, correction of retention time, and extraction of peak area. The data obtained from XCMS processing were combined with retention time and *m/z* data to identify each metabolite. The processed data was imported into SIMCA-P software for further analysis (v14.0, Umetric, Umea, Sweden). After being log10 transformed, Par scaled was performed on the peak intensity data, and then principal component analysis (PCA), partial least squares-discriminate analysis (PLS-DA), and orthogonal to partial least squares-discriminate analysis (OPLS-DA) were undertaken. A permutation test (200 permutations) was performed to evaluate the fitness of the OPLS-DA model. The VIP values generated by OPLS-DA were used to identify potential biomarkers between two groups. A two-tailed student *t* test was used for statistical comparison, and *P* < 0.05 was considered statistically significant. The selection standard of significant differential metabolites (SDMs) was VIP >1 and *P* < 0.05.The identified metabolites were mapped to the KEGG database using KEGG mapper (26). KEGG pathway enrichment analysis of differential metabolites was analyzed by MSEA (Metabolite Sets Enrichment Analysis) (27). The significance was determined by hypergeometric test, and the corrected *P* < 0.05 was set. A Venn diagram was drawn using the web application: http://jvenn.toulouse.inra.fr/app/ index. html.

### Fuzzy c-means cluster analysis

The peak intensity values of differential metabolites were clustered using Fuzzy c-means clustering from Mfuzz v2.42 R package (28). Only metabolites with significant differences in peak intensity between pairwise age group (VIP>1, *P*-value <0.05) were used as input for this clustering analysis. The optimal number of clusters was set to three.

### Weighted gene co-expression network analysis

A weighted gene correlation network analysis (WGCNA) was performed on the log-transformed metabolite abundances matrix using the WGCNA package in R (29). This analysis generates a correlation matrix between all differential metabolites and age to identify groups of metabolites that are highly related. The relationships between the module eigengene (ME) gene sets and the clinical traits (age) were evaluated with gene significance (GS) and module membership (MM). The hub metabolites were then screened by combining MM>0.8 and GS>0.2.

### Fecal DNA isolation and 16s rRNA sequencing

Total DNA of the fecal samples was extracted using the Cetyltrimethylammonium Bromide (CTAB) method. Concentration of the DNA samples was monitored on 1% agarose gels. DNA was diluted to 1 ng/µL using sterile water according to the concentration. Amplification and sequencing of the V3–V4 region were performed using the universal primers 341 F (5′-barcode- ACTCCTACGGGAGGCAGCA -3′) and 806 R (5′- GGACTACHVGGGTWTCTAAT -3′) (30). All PCR reactions were undertaken with Phusion High-Fidelity PCR Master Mix (New England Biolabs). The PCR products were pooled, confirmed by 2% agarose gel electrophoresis, and purified with Qiagen Gel Extraction Kit (Qiagen, Germany). Sequencing libraries were generated using TruSeq DNA PCR-Free Sample Preparation Kit (Illumina, USA) following the manufacturer’s recommendations and index codes were added. The library quality control checks and quantification were performed on the Agilent Bioanalyzer 2100 system (Agilent Technologies, Santa Clara, CA). Lastly, sequencing was performed on Illumina NovaSeq 6000 (Illumina Inc., San Diego, CA, USA).

### 16s rRNA sequencing analysis

Reads were analyzed using QIIME2 version 2020.2 after truncating the barcode and primer sequences (31). The denoising process and clustering into amplicon sequence variants (ASVs) were performed by DADA2 plugin (32). Taxonomic assignment was conducted against greengenes13_8 99% ASV reference sequences (http://greengenes.lbl.gov/cgi-bin/nph-index.cgi). Samples were rarefied at a threshold of minimum sequence frequency in all samples. The ASV abundance table was normalized, and ASVs were collapsed at the generic level to obtain a nonredundant species catalog table.

At an even sampling depth of 22,504 sequences per sample, alpha diversity was calculated using R to calculate the Shannon index, richness index, evenness index, and Faith’s Phylogenetic Diversity (Faith-pd) index, and the statistical significance between groups was determined using Kruskal-Wallis tests with post hoc Wilcoxon rank sum tests. Beta diversity measuring the differences between samples was based on Bray-Curtis distances using the R “vegan” package. We used PERMANOVA to test the differences of beta diversity between groups (33). LEfSe was used to identify significant differences in bacteria abundance between groups (34). Linear discriminant analysis (LDA) scores greater than 2.0 were considered as significant. Data visualization was conducted in R using vegan (35) and ggplot2 packages (36). The wardD2 algorithm using Euclidian distances was used to perform clustering. Spearman correlation analysis was performed on the differential metabolites and differential flora in all groups using R corrplot (37).

### Metagenome sequencing

Next generation sequencing library preparations were constructed following the manufacturer’s protocol (VAHTS Universal DNA Library Prep Kit for Illumina). The fecal samples from Cubs, Young, Adults, and Old pandas (three samples per group) were analyzed where 200 ng genomic DNA was randomly fragmented to <500 bp by sonication (Covaris S220) with the setting parameters of peak power: 175 w, duty factor: 10%, Cycles/Burst: 200, and times: 50 s. The fragments were treated with End Prep Enzyme Mix for end repairing, 5’ Phosphorylation and dA-tailing in one reaction, followed by a T-A ligation to add adaptors to both ends. Size selection of Adaptor-ligated DNA was then performed using VAHTSTM DNA Clean Beads, and fragments of ~470 bp were recovered. Each sample was then amplified by PCR for eight cycles using P5 and P7 primers, with both primers carrying sequences that can anneal with flowcell to perform bridge PCR and P7 primer carrying a six-base index allowing for multiplexing. The PCR products were validated using an Agilent 2100 Bioanalyzer (Agilent Technologies, Palo Alto, CA, USA) quantified by Qubit 3.0 Fluorometer (Invitrogen, Carlsbad, CA, USA), and sequenced using an Illumina HiSeq/Novaseq instrument according to manufacturer’s instructions (Illumina, San Diego, CA, USA).

### Metagenomic analysis

From the raw metagenomic reads, the Illumina adapters were removed using the KneadData (v0.5.1) toolkit (38) and reads aligned to the giant panda genome were removed using KneadData integrated Bowtie2 tool (version 2.3.4.1) (39). The quality of the metagenomes was then tested using the FastQC toolkit (version 0.11.7) (40). Taxonomic composition of the metagenomes was profiled by Kraken2 (41). After the scaffold sequences from all samples were assembled by Megahit (version 1.1.3) (42), prodigal (version 3.02) (43) was used to predict open reading frames (ORFs) and translated into amino acid sequences. Cd-Hit (version 4.5.6) (44) was applied to build nonredundant gene sets for all predicted genes with more than 95% identity and more than 90% coverage. The gene with the longest full length from each cluster was selected as the representative read of each gene set. For further analysis, function annotations were performed with the gene set representative reads using Blastp (Blast version 2.2.28+) (45) alignment (E-value < 0.00001) between ORFs and the protein databases of the Kyoto Encyclopedia of Genes and Genomes, CAZy (http://www.cazy.org/) and CARD (https://card.mcmaster.ca/). In addition, profiling of genes encoding microbial biochemical pathways was performed using the HUMAnN2 pipeline (version 0.11.1) (46) integrated with the DIAMOND alignment tool (version 0.8.22) (47), uniref90 protein database (version 0.1.1) (48), and the ChocoPhlAn database (version 0.1.1) (49). The Kruskal-Wallis (K-W) test (50) and Benjamini and Hochberg of multiple testing (51) were performed to identify CAZymes that were significantly different between different age groups with the significance setting of *P*-value <0.05. Analysis of CAZymes with significant differences between two aged group pairs was also conducted using STAMP (52).

## RESULTS

### Age-associated changes in fecal metabolome

**Fig 1:**
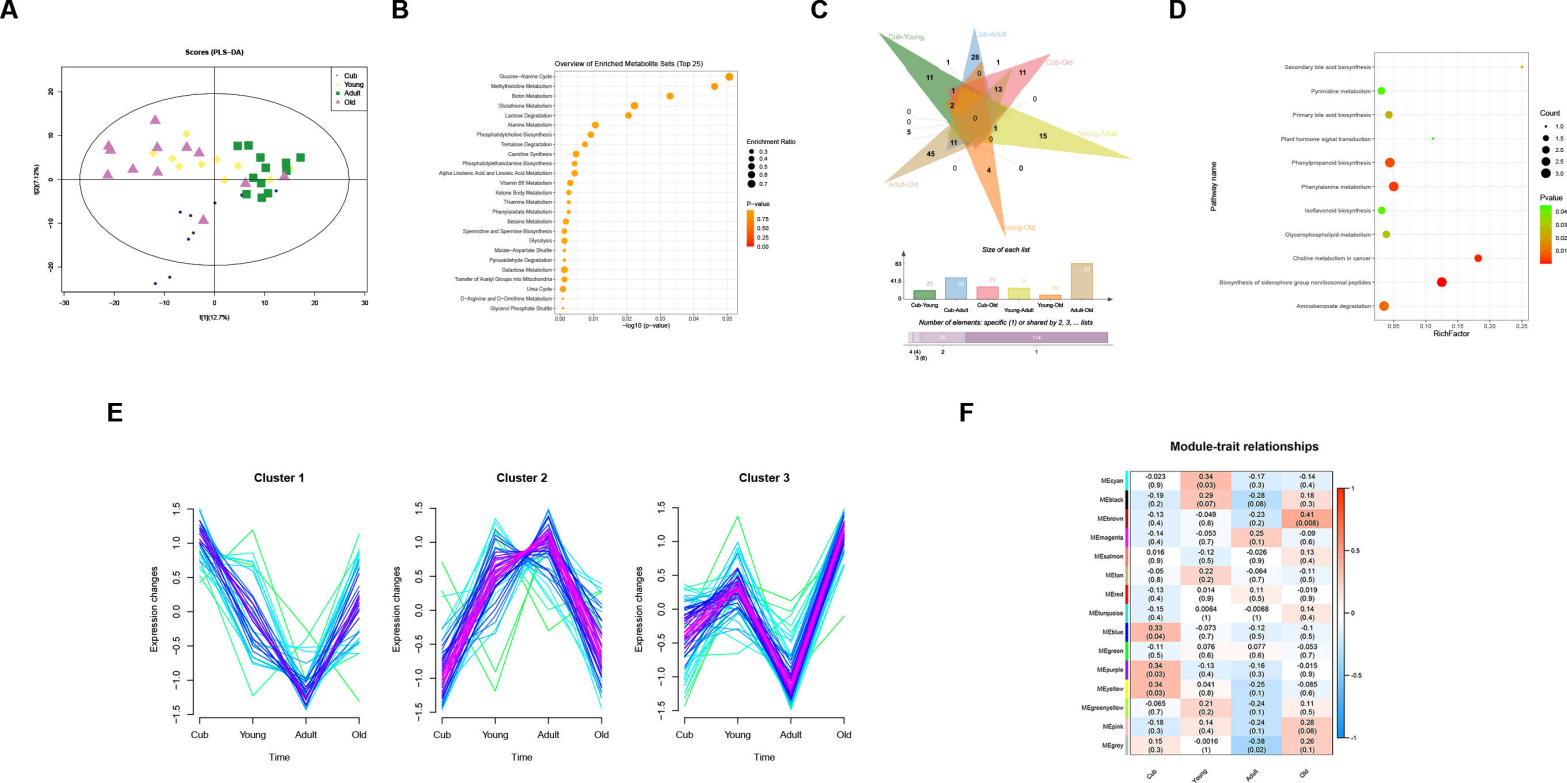
(**A**) PLS-DA score plots of fecal metabolites between the different age groups. (**B**) All metabolites annotated in KEGG database in the top 25 significant pathways. (**C**) Venn diagram of the numbers of SDMs in any two-comparison groups. (**D**) KEGG enrichment results of all SDMs. (**E**) Cluster analysis of SDMs. (**F**) Correlation heatmap between modules and different age groups.

A total of 837 and 539 metabolites were identified by UPLC-Q-TOF in positive and negative ion modes, respectively. A supervised method, OPLS-DA, was performed to evaluate the separation between the four age groups. Different age groups were clearly differentiated from each other in both ESI modes (Fig. S1). PLS-DA results showed that the four groups were well separated (Fig. 1A). These results indicated that the metabolic profiles significantly differed between the four age groups of giant pandas. KEGG annotation of all identified 1,376 metabolites indicated that they were mainly involved in amino acid metabolism (e.g., alanine cycle, histidine metabolism, and alanine metabolism), lipid metabolism (e.g., the biosynthesis of phosphatidyl choline, carnitine synthesis, and metabolism of linoleic acid), and carbohydrate metabolism (e.g., lactose degradation, trehalose degradation, and metabolism of galactose and glycolysis) (Fig. 1B). These metabolites are involved in the growth and development of giant pandas.

We identified upregulated and downregulated metabolites to compare the concentrations of metabolites in different age groups. The age-related SDMs were defined as VIP > 1 and *P* < 0.05, and 20, 50, 29, 26, 10, and 83 SDMs were identified between the Cub vs Young, Cub vs Adult, Cub vs Old, Young vs Adult, Young vs Old, and Adult vs Old comparisons, respectively. These SDMs included 152 metabolites that were identified as differentially expressed in at least one of the comparisons. We also found that the vast majority (97/67%) of SDMs were only found to be differentially expressed in one comparison group, and no SDM was shared by all comparative groups (Fig. 1C).

We found that the 20 SDMs in the Cub vs Young comparison comprised 7 upregulated and 13 downregulated SDMs in the Cub when the relative intensity of metabolites was assessed. The contents of Desferrioxamine were significantly higher in Cub samples, while the contents of 4-hydroxybenzoate and D-psicose were significantly higher in Young samples (Table S2). The 50 SDMs between Cub vs Adult were comprised of 24 upregulated and 26 downregulated SDMs in Cub samples. The 1,2-dimyristoyl-sn-glycero-3-phosphate showed the greatest upregulation (Fold change = 32.57) and the Flavone base+3o, 2Meo, o-guaiacylglycerol showed the greatest downregulation (Fold change = 0.01) in Cub samples (Table S2). We found that metabolites such as coumarin, salicylic acid, and syringic acid were downregulated in Cub samples, and these metabolites are bamboo extracts (53). Metabolites of Acetylcarnitine and Choline were significantly upregulated in Cub samples. We found 29 SDMs in the Cub vs Old comparison, with 4 upregulated and 25 downregulated SDMs in Cub samples. The contents of Leu-Gln-Arg and Heptadecasphinganine were significantly higher in Cub samples, while the contents of Tauroursodeoxycholic acid, Taurodeoxycholic acid, and Taurochenodeoxycholic acid were higher in Old samples (Table S2). A total of 26 SDMs were obtained in the Young vs Adult comparison. The contents of Arachidonoylserotonin, Leukotriene d4 methyl ester, and Hypoxanthine were significantly higher in Young samples, while the contents of 3-hydroxyisovaleric acid, deoxycarnitine, and N-acetylglucosaminylasparagine were significantly higher in Adult samples (Table S2). The SDMs in the Young vs Old were eight upregulated SDMs and two downregulated SDMs in Young samples. Of these, Brefeldin A (Fold change = 4.83) was the largest difference in upregulated SDMs, and Ligustroflavone (Fold change = 0.03) was the largest difference in downregulated SDMs (Table S2). There were 83 SDMs in Adult vs Old comparison, with 27 upregulated and 56 downregulated metabolites in Adult samples. The contents of 1,7-dimethyluric acid, pyridoxine, and deoxycarnitine were higher in the Adult samples, whereas taurodeoxycholic, tauroursodeoxycholic acid, glycodeoxycholic acid, chenodeoxycholate acid, Lpe 18:2, and choline were higher in the Old samples (Table S2).

KEGG enrichment analysis was performed on all identified SDMs, which were enriched in 20 pathways, including Biosynthesis of siderophore group nonribosomal peptides, Phenylalanine metabolism, Phenylpropanoid biosynthesis, Aminobenzoate degradation, Secondary bile acid biosynthesis, Primary bile acid biosynthesis, Glycerophospholipid metabolism, Isoflavonoid biosynthesis, Pyrimidine metabolism, and Plant hormone signal transduction (Fig. 1D). Our results indicated that these metabolic pathways were significantly different between at least two or more stages.

### Trend analysis of age-related metabolites

A trend analysis was conducted on the 152 identified SDMs by MFuzz software. The 152 SDMs were grouped into three clusters according to metabolite intensity (Fig. 1E). Metabolites in Cluster 1 had the highest abundance in Cub samples, then their relative abundances decreased from Cub to Adult and recovered in Old samples. Cluster 1 comprised 36 SDMs, such as kynurenic acid, hippuric acid, uracil, Lpe 18:2, acetylcarnitine, choline, and hypoxanthine, which were enriched in nucleotide and amino acid metabolism pathways, including pyrimidine metabolism, purine metabolism, and tryptophan metabolism (Table S3). Cluster 2 contained 52 SDMs, and these SDMs increased from Cub to Adult samples and were relatively high in Young and Adult samples, then decreased in Old samples. Metabolites 1,7-dimethyluric acid, pyridoxine, deoxycarnitine, and 4-hydroxybenzoate were in Cluster 2, and they were mainly enriched in vitamin metabolism pathways. Cluster 3 contained 64 SDMs, and their contents fluctuated from Cub to Adult and were at their lowest level in Adult samples and highest in Old samples. Cluster 3 included metabolites such as taurochenodeoxycholic acid, taurodeoxycholic acid, chenodeoxycholate, tauroursodeoxycholic acid, glycodeoxycholic acid, citrate, and Fahfa 36:2. Many metabolites (33%) involved in protein and amino acid metabolism were significantly enriched in Old samples, such as dipeptides (i.e., Glu-Leu, Asp-Glu, Gln-val, and His-Leu) and tripeptides (i.e., Gly-Cys-Arg, Gln-Phe-Arg, Leu-Gly-Leu, and Asp-Leu-Arg). This suggested decreases in incomplete protein catabolism or absorption in the Old samples. SDMs in Cluster 3 were mainly enriched in digestive metabolism and lipid metabolism pathways (Table S3).

### Age-related hub metabolites

A WGCNA was performed to identify metabolites within age groups. By calculating the pairwise correlations between all metabolites in the dataset, the metabolites were assigned to 15 modules. Of these, six modules showed significant module-trait correlations with a particular age, with *P* < 0.05 (Fig. 1F). The module Blue (*r* = 0.33, *P* = 0.04), Purple (*r* = 0.34, *P* = 0.03), and Yellow (*r* = 0.34, *P* = 0.03) were significantly positively correlated with Cub samples. There were 145 metabolites including 6 SDMs in the Blue module, 54 metabolites including 5 SDMs in the Purple module, and 121 metabolites including 5 SDMs in the Yellow module. The Cyan module contained 38 metabolites and was significantly positively correlated with Young samples (*r* = 0.34, *P* = 0.03). Metabolites in the Grey module showed a significant negative correlation with Adult samples (*r* = −0.38, *P* = 0.02) and contained 120 metabolites (including 14 SDMs). The Brown module showed a significant positive correlation with Old samples (*r* = 0.41, *P* = 0.08), and there were 126 metabolites in the module including 28 SDMs (Table S4).

Hub metabolite analysis of the significant trait-correlation modules was used to identify the most important metabolites related to a particular age group. We identified 28, 25, 48, 10, and 39 hub metabolites in the Blue, Purple, Yellow, Cyan, and Brown modules, respectively, while no hub metabolite was identified in the Grey module. There were 101 hub metabolites with a strong positive correlation with Cub samples, with 10 being SDMs. Ten hub metabolites were positively correlated with Young samples and 39 hub metabolites including 6 sDMs were positively correlated with Old samples (Table 1).

**TABLE 1:** The information of significance modules with hub metabolites

Module	Correlation age	No. of hub metabolites	No. of DEMs in hub metabolites	Name of DEMs in hub metabolites
Blue	Cub	28	0	\ ^a^
Purple	Cub	25	4	2-O-methylguanosine, hypoxanthine, Pro-Hyp, and hippuric acid
Yellow	Cub	48	6	Hydroxyquinoline, alisol a 24-acetate, atorvastatin, bis, 1,1 '- (1,8-dioxo-1,8-octanediyl) [glycyl-glycine], and kynurenic acid
Cyan	Young	10	0	\
Brown	Old	39	6	Asp-glu, Gln-Phe-Arg, Gly-Pro-Glu, Methyl(1 s)-7-hydroxy-7-methyl-1-[(2 s,3r,4s,5s,6r)-3,4,5-trihydroxy-6-(hydroxymethyl) oxan-2-yl], coxy-4a,5,6,7a-tetrahydro-1h-cyclopenta[c]pyran-4-carboxylate, His-Leu, and Pro-Glu

^*a*^ \ represents no DEMs in hub metabolites

### Age-associated changes in microbial biodiversity and composition

**Fig 2:**
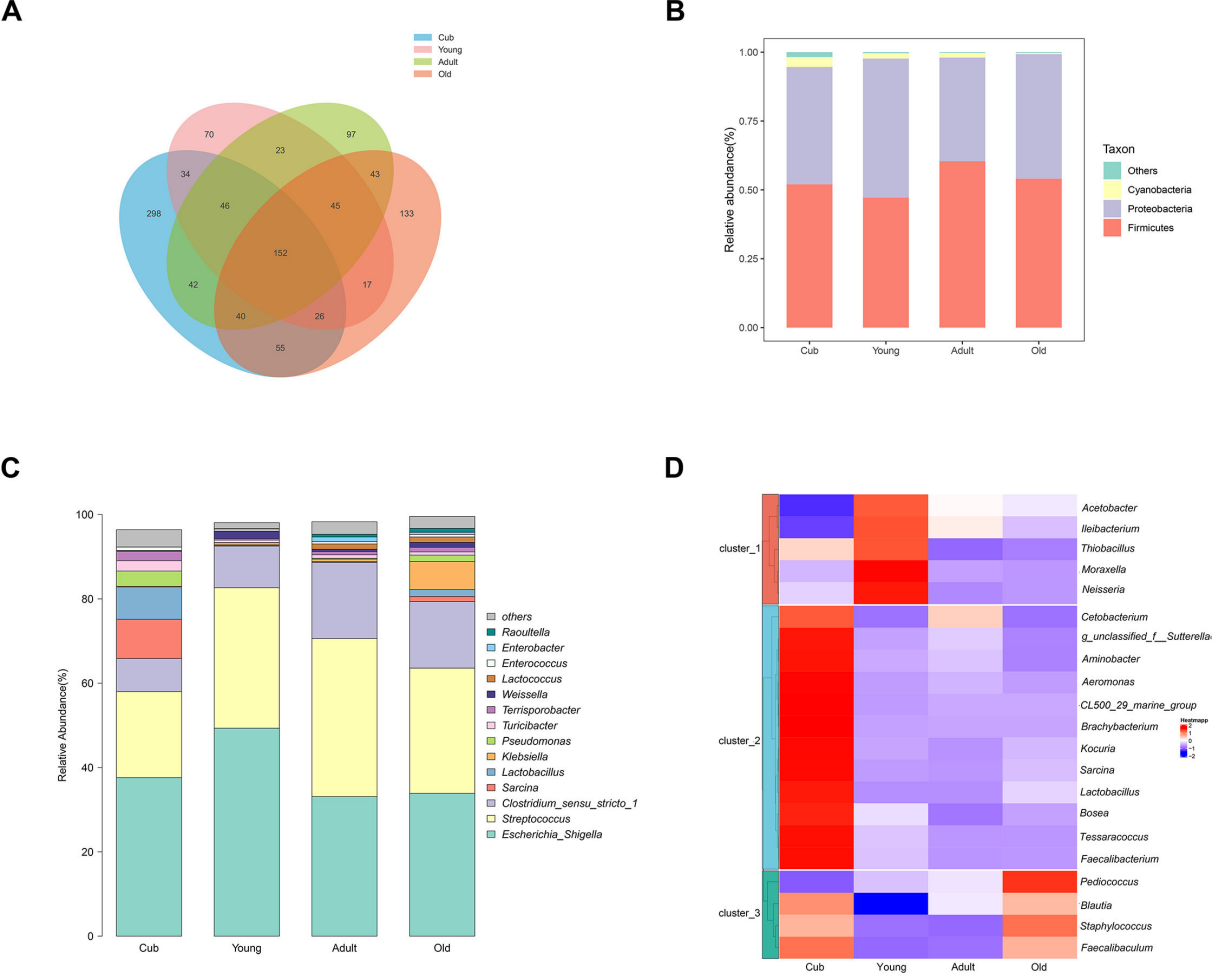
(**A**) Venn diagram of ASVs number in different age groups. (**B**) Relative gut microbiota abundance at the phylum level in the different age groups. (**C**) Relative gut microbiota abundance at the genus level in the different age groups. (**D**) The cluster heatmap of relative abundance of genus with significant differences among four age groups. The abundance of significant differential bacteria (SDB) was log transformed and was indicated as different colors from cold color to warm color in the heatmap.

The 16S rRNA genes in fecal samples from the four age groups were amplified and sequenced to examine variations of gut microbiota. We obtained 79,266–134,355 valid reads from 44 fecal samples, with an average of 113,993 sequences per sample. After filtering, analyses generated 1,121 ASVs. The sobs dilution curve and species richness showed that the sample size provided enough sequences to measure bacterial variation (Fig. S2A and B). We used classification annotation and found that 18 phyla and 264 genera were annotated from 1,121 ASVs. There were 693, 413, 488 and 511 unique ASVs in the Cub, Young, Adult, and Old groups, respectively, with the highest number of unique ASVs in the Cub group (Fig. 2A).

We found that the α-diversity evenness indices of Ace (Fig. S2C), Shannon (Fig. S2D), and Simpson (Fig. S2E) and the richness index of Faith-Pd (Fig. S2F) decreased in order of age, from Cub to Old samples. However, only the Shannon index significantly decreased from Cub to Adult and Cub to Young. Supervised PLS-DA clustering analysis showed that the four age groups were well separated, and the Cub was the most distinct group from other age groups with respect to gut microbiota (Fig. S2G).

Analysis of microbial composition indicated that it was dominated by phyla Firmicutes and Proteobacteria (Fig. 2B). *Streptococcus*, *Escherichia/Shigella*, *Clostridium_sensu_stricto_1*, *Sarcina*, and *Lactobacillus* were the dominant genera, but their percentages varied between the age groups (Fig. 2C). *Lactobacillus* was dominant in the Cub group unlike the other age groups and maybe related to cubs being exclusively fed breast milk and/or formula. Additionally, the relative abundance of *Clostridium_sensu_stricto_1* was highest in the Adult but lowest in the Cub group.

Differences of microbial composition at the genus level were then compared using Kruskal-Wallis tests. We identified 21 significant differential genera (*P* < 0.05) and a hierarchical heatmap illustrated that the abundance of the 21 genera significantly differed between age groups (Fig. 2D). *Faecalibacterium*, *Sarcina*, *Aeromonas*, *Aminobacter*, *Cetobacterium*, *Tessaracoccus*, *Bosea*, *Brachybacterium*, and *Staphylococcus* were significantly more abundant in the Cub group. The relative abundance of *Moraxella*, *Neisseria*, *Ileibacterium*, and *Kocuria* was significantly greater in the Young group, while *Pediococcus* was significantly greater in the Old group.

### Metagenome-based functional profiles between the four age groups

**Fig 3:**
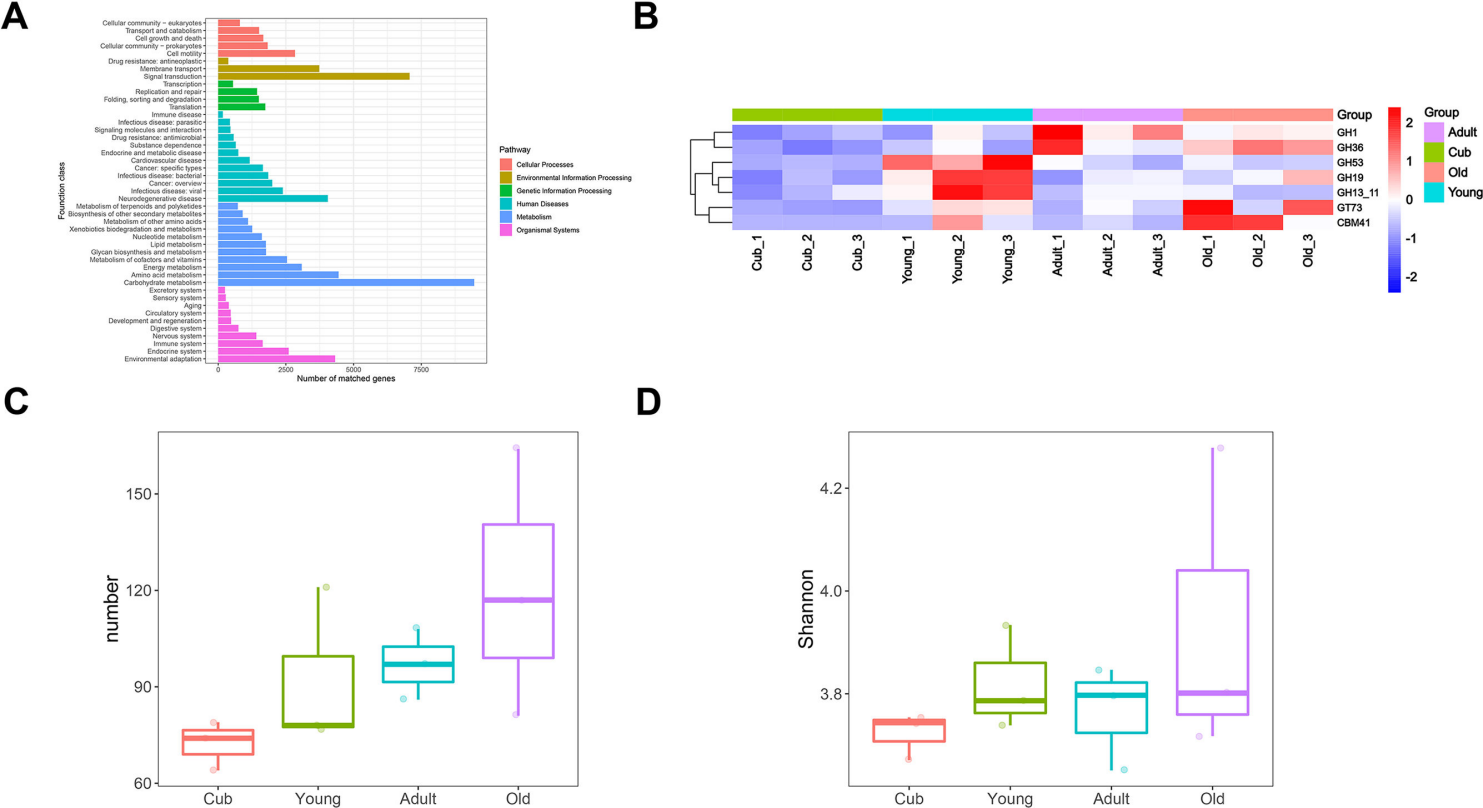
(**A**) KEGG level two pathway annotation results of all samples. (**B**) Heatmap of different CAZyme between groups (*P* < 0.05). The relative abundance of significant differential cazyme is log transformed and is indicated as different colors from cold color to warm color in the heatmap. (**C**) Boxplot of ARG numbers in each group. (**D**) Boxplot of ARG’s diversity in each group.

We sampled three individuals from each age group and performed a metagenome analysis to investigate the functional profile of giant panda gut microbiomes across the four age groups. A total of 71,405.82 Mbp of raw reads were obtained from the metagenomic sequencing with an average sequencing data of 5,950.49 Mbp per sample. The effective data rate of quality control was 99.45% (Table S5). All the assembled metagenome data were annotated by KEGG. The highest number of genes was associated with carbohydrate metabolism on the second metabolic pathways level (Fig. 3A). This suggests that carbohydrates are the main energy source of the giant panda gut microbiome. LEfSe analysis further identified functional changes in gut microbiome metabolism between the age groups. Our analysis showed that methane metabolism and seleno compound metabolism pathways were enriched in the Old group. The relative abundance of base excision repair and a foxO signaling pathway were enriched in the Adult group. The Cushing syndrome pathway was enriched in the Cub group (Fig. S3A).

Analysis of the CAZyme profiles of the gut microbiome identified 277 CAZymes genes across the four age groups. Matched gene numbers of category GH (glycoside hydrolases) were the greatest (139), with GH24, GH1, GH23, GH4, GH3, and GH2 being the dominant CAZymes in the GH family (Fig. S3B). Among them, GH1, GH3, and GH2 belong to cellulose degrading enzymes, which may greatly contribute to cellulose degradation in the giant panda. We identified seven CAZymes with significantly different abundances between groups (Fig. 3B) using the Kruskal-Wallis and Benjamini and Hochberg test (*P* <0.05). Of these seven, GH1 is involved in cellulose degradation and was highly enriched in Adult and Old groups. GH53, GH19, and GH13_11 were enriched in the Young group, and they participate in hemicellulose and amylase degradation. CBM41 and GT73 were more abundant in the Old group. All of these CAZymes had relatively low abundances in the Cub group, indicating carbohydrate metabolism in the Cub group was significantly different to older panda age groups. Meanwhile, STAMP analysis indicated that the relative abundances of 27 CAZymes were significantly different between pairs of adjacent age groups (*P* < 0.05), with 7 CAZymes significantly greater in the Young than in the Cub group, 14 CAZymes significantly greater in the Young than in the Adult group, and only 5 CAZymes significantly greater in the Old than in the Adult group (Fig. S3D). Both the Kruskal-Wallis tests and the STAMP analysis identified GH13_11, GH53, and GH19. Of these, GH13_11 and GH53 are related to hemicellulose degradation.

We found 237 different types of antibiotic resistance genes (ARGs) in all samples, with the highest ARG number (113) in the Old group and the lowest number (66) in the Cub group. The top 20 most abundant ARGs were selected to construct the heatmap, which indicated that their abundance varied between the four age groups (Fig. S3C). The Old group had more multidrug resistance genes, and the number and diversity of ARGs increased with age group (Fig. 3C and D).

Metagenomic functional profiling was analyzed using HUMAnN2, and the pathway abundances were compared using LEfSe. In total, 88 metabolic pathways varied between the age groups (LDA score >2, Kruksal-Wallis test, Benjamini-Hochberg FDR, *q* < 0.05; Fig. S4). The Young group was enriched in pathways associated with carbohydrate metabolisms, such as starch degradation, gluconeogenesis, and pentose phosphate pathways, while pathways associated with coenzyme A biosynthesis and heme biosynthesis were more abundant in the Cub group. However, pathways associated with energy production were more abundant in the Adult group, such as dTDP biosynthesis, NAD biosynthesis, and ppGpp biosynthesis. Pathways involved in degradation processes such as acetylene degradation, glycerol degradation, glycogen degradation, and L histidine degradation, as well as palmitate biosynthesis, and L methionine biosynthesis were abundant in the Old group.

**Fig 4:**
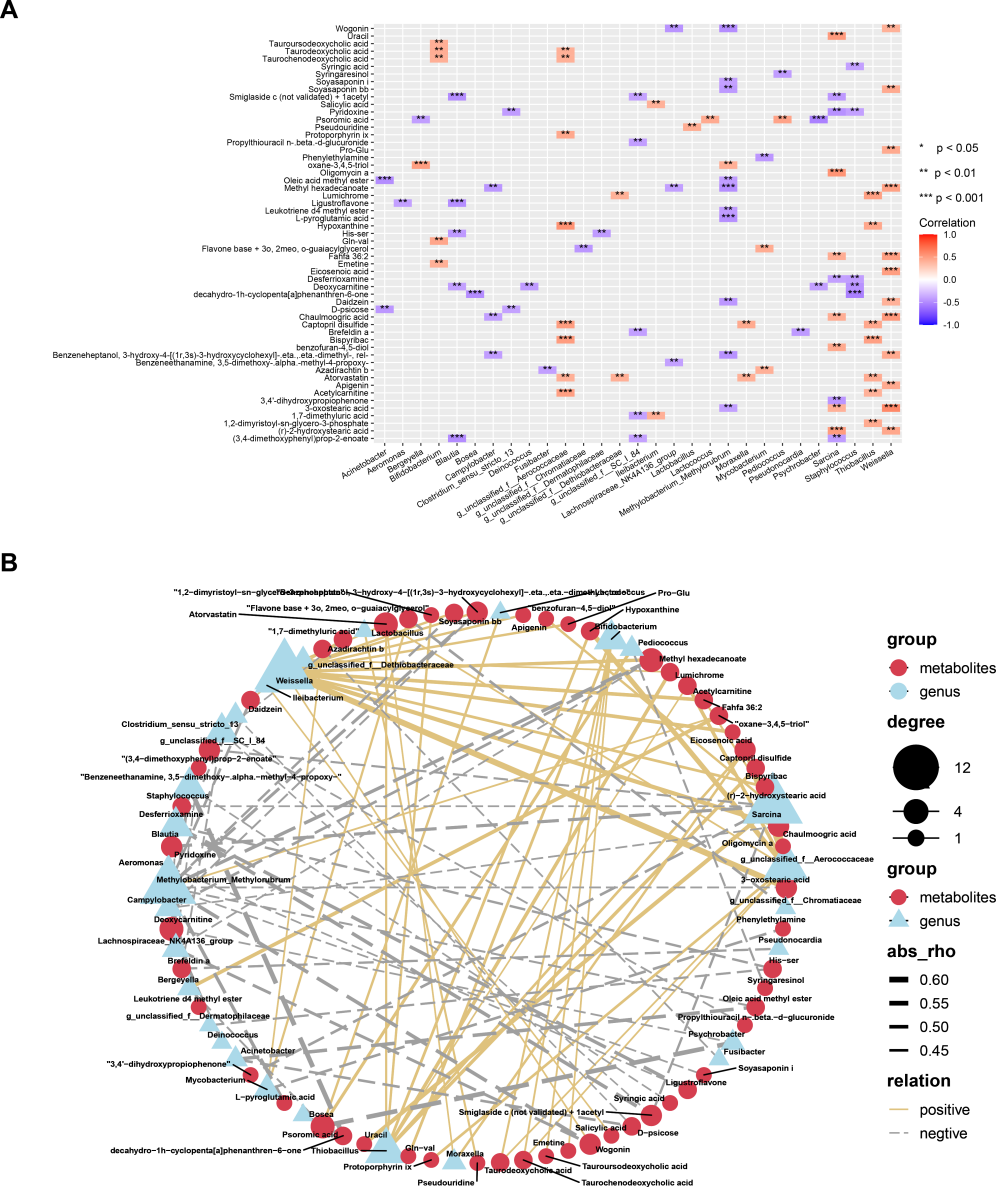
(**A**) Heatmap summarizing the correlation of altered gut microbiota genera and fecal metabolites between the different age groups. (**B**) Network correlation between SDMs and differential genus (*P < 0.05, **P < 0.01, and ***P < 0.001).

### Correlations of the age-related differential microbiota and SDMs

A correlation matrix was generated by calculating Spearman’s correlation coefficient to explore any correlation between SDB and SDMs. Twenty-nine genera of SDB and 55 SDMs were correlated (coefficient *r* > 0.4; Fig. 4A). We found 467 significant associations (*P* < 0.05), with 188 positive correlations and 279 negative correlations between the SDB and SDMs (Table S6). Network analysis confirmed the correlations between SDMs and SDB with a related coefficient of *r* > 0.4 (Fig. 4B). We found many bile acid related SDMs had significant correlations with several SDBs. For example, taurochenodeoxycholic acid, taurodeoxycholic acid, glycodeoxycholic acid, tauroursodeoxycholic acid, and chenodeoxycholate were significantly positively correlated with *Bifidobacterium, Lactobacillus, and Weissella*, while most had significant negative correlations with *Faecalibacterium. Sarcina*and *Weissella* had the most correlations with the age-related SDMs. *Sarcina* was significantly positively correlated with seven SDMs, including uracil, oligomycin A, chaulmoogric acid, 3-oxostearic acid, r-2-hydroxystearic acid, and Fahfa 36:2 and was significantly negatively correlated with five SDMs, including pyridoxine, desferrioxamine, and 3,4'-dihydroxypropiophenone. *Weissella* was positively correlated with 12 SDMs, including Fahfa 36:2, Pro-Glu, chaulmoogric acid, eicosenoic acid, 3-oxostearic acid, and r-2-hydroxystearic acid.

## DISCUSSION

### Characterization of metabolite profiles in the giant panda

Giant pandas exhibit distinct metabolic characteristics with relatively low basal metabolic rate and low energy metabolic rate compared to similar sized mammals (54, 55). However, how the physiological metabolic characteristics affect the content of metabolites in different developmental stages of giant pandas is less understood. The present study performed untargeted UPLC-Q-TOF-MS metabolomics analysis based on 44 captive giant pandas in four age groups and identified 1,376 metabolites involved in various aspects of growth and development. However, in the previous studies on the metabolome of giant pandas, only 400 metabolites were identified in the feces of giant pandas using LC-MS (22) and 107 metabolites using 1 h NMR (21). We identified considerably more metabolites, and this can better represent the metabolic profile of giant pandas at different age stages.

### Metabolomics and microbiota community analysis provides new insights into the giant panda diet transition

Like all mammals, giant pandas transition from a milk/formula-only diet in cubs to a non-milk-based diet when they are weaned, but unlike other carnivores they transition to herbivory in adults. We found that metabolites and gut microbiota communities changed significantly in response to this dietary transition. Our results showed that there were 152 SDMs across the four giant panda age groups (i.e., Cub (1.1, 2 yr), Young (3, 7 yr), Adult (11, 18 yr), and Old (20, 28 yr)). SDMs in the Cub group had higher active lipid metabolism than the older age groups. The upregulated SDMs in the Cub group were mainly enriched in phospholipid biosynthesis and oxidation pathways of branched fatty acids. We also found lipid metabolites, such as choline, acetylcarnitine, and LysoPC 16:0, had higher abundances in the Cub group. Choline is an essential nutrient and is mainly consumed through the diet, existing in a variety of foods including formula (56). In addition, hippuric acid was identified to be a hub metabolite in the Cub group and its elevated levels suggested that it was associated with the consumption of a fat-rich diet (57). Mfuzz clustered these lipid metabolites to Cluster 1 with the highest abundance in the Cub group and they declined with age groups. Similarly, the concentration of total fatty acids in human newborn babies’ feces was significantly different between a diet based on breast milk and formula (58). Interestingly, we found several lipid metabolites that were higher in the Old group compared to the Young and Adult groups. We hypothesize that these higher lipid metabolites in old pandas maybe related to the aging caused disorder in lipid metabolism but this will require further study.

After weaning, young giant pandas transit into a bamboo-rich diet. Correspondingly, we identified that many plant secondary metabolites from our metabolome data were significantly higher in the Young and Adult groups compared to the Cub group, such as deguelin, coniferyl aldehyde, eriodictyol, flavonol base + 4o, o-hex-dhex-pen, and flavone base +3o,2meo, o-gua acylglycerol. Mfuzz analysis clustered these plant secondary metabolites to Cluster 2. Flavonoids are important plant secondary metabolites and are widely found in bamboos (59). Metabolomic analysis has shown that dietary bamboo of wild and captive giant pandas contains multiple types of flavonoids (60). We also found the isoflavone biosynthesis pathway was enriched in the Young and Adult groups, which was consistent with Yang et al.’s results (22). Other bamboo extract-related molecules like syringic acid, salicylic acid, and coumarin were also significantly higher in the Adult group than in the Cub group. The large intake of bamboo in the Young and Adult individuals contributed to the increase in the level of related metabolites.

Changes in the host’s diet can affect the composition and diversity of gut microbiota (61).We found that the α-diversity of the microbiota community was lower in giant pandas that ate exclusively bamboo, based on 16S sequencing data. The highest α-diversity was in the Cub group, followed by the Young group and was the lowest in the Adult group. Zhang et al. (62) and Guo et al. (63) also found that the largest abundance of ASVs in the gut microbiota was prior to giant pandas consuming bamboo, and gut microbiota diversity significantly decreased after living on a bamboo-based diet. This lower α-diversity in the gut microbiome suggests two possible, not mutually exclusive, causes. Firstly, the simpler and more specialized bamboo-eating diet in adulthood compared to cubs results in a simpler composition. Secondly, the antimicrobial components that exist in their bamboo (63) suppress bacteria proliferation.

We also found that bacteria related to the digestion of cellulose were significantly more abundant in adult pandas. Many bacteria in Firmicutes such as *Enterococcus* and *Lactococcus* are thought to contribute to lignocellulose digestion in giant pandas (64). In fact, *Enterococcus*, *Lactococcus,* and Firmicutes were significantly more abundant in the Adult group (Firmicutes: 60.42% ± 0.25) than in the Cub (Firmicutes: 52.02% ± 0.26). In addition, other bacteria related to a high-fiber diet were more abundant in the Adult than the Cub group, such as *Streptococcus* increasing from 20.40% ± 0.29 (Cub) to 33.62% ± 0.15 (Adult), and *Clostridium* increasing from 7.86% ± 0.19 (Cub) to 18.07% ± 0.08 (Adult). *Streptococcus* may protect the intestine from damage caused by a high-fiber content and promote the movement of high-fiber components in the intestine, which is crucial for the giant panda’s transition from a low-fiber to a high-fiber diet (65). *Streptococcus* has the potential to degrade cellulose in the intestinal tract of giant pandas (66). *Clostridium* was positively correlated with the digestibility of crude fiber and can produce genes encoding cellulose or hemicellulose degradation enzymes. Consistent with our results, previous studies have found that the relative abundance of *Streptococcus* and *Clostridium* significantly increased with increasing bamboo content in the diet of giant pandas (64, 67).

We found that the genes involved in carbohydrate metabolism were the most abundant in our functional annotation of the metagenome. In particular, we identified 277 CAZyme genes, including GH1, GH3, and GH2, that play important roles in cellulose degradation and were the dominant CAZymes in the GH family of the giant panda metagenome. GH1 and GH3 provide genes encoding β-glucosidase that plays a major role in the degradation of hydrolyzing cellobiose or cello-oligosaccharides to glucose. Both GH1 and GH3 are the key enzymes in the metabolic pathway of cellulose degradation (68). The dominance of these CAZymes suggests that giant panda has the potential to degrade bamboo cellulose through its gut microbes. The giant panda possesses a digestive tract typical of carnivores, lacking the enzyme homologs involved in cellulose digestion (69), therefore, CAZymes related to cellulose digestion in the gut microbiota are important for giant pandas. We then identified CAZymes that were significantly different between age groups, which yielded 7 and 27 differential CAZymes using Kruskal-Wallis tests and STAMP analysis, respectively. GH13_11, GH53 and GH19 were identified as being significantly different by both methods, and GH13_11 and GH53 are related to hemicelluloses degradation. Interestingly, the abundances of the seven CAZymes were lowest in the Cub group (Fig. 3B). The abundance of GH13_11 that encodes hydrolytic amylase (70) was greater in the Young group, and GH1 that encodes cellulose degrading enzyme (70) was greater in the Adult and Old groups. We also found that the Young group had considerably more differential CAZymes (21 CAZymes, Fig. S4) and all were more abundant in the Young group than either the Cub or the Adult group (Fig. 3B; Fig. S4). Our results suggest that gut microbiota in cubs change significantly in response to the diet transition from high protein, low cellulose milk to high cellulose, low starch bamboo in young pandas, especially highly functioning carbohydrate degradation in young panda gut microbiota.

As the diet transitions, the microbiota community also undergoes changes in the abundance of beneficial and pathogenic bacteria. Our study found that beneficial bacteria, such as *Faecalibacterium*, *Sarcina*, and *Blautia,* were abundant in the Cub group and rarer in the Young group. In contrast, several potential pathogenic bacteria, such as *Moraxella* and *Neisseria*, were found enriched in the Young group but not in the other age groups. *Faecalibacterium* (71), *Sarcina* (72), and *Blautia* (73) are short-chain fatty acid (SCFA)-producing bacteria. *Faecalibacterium* is involved in butyrate synthesis, which is the main nutrient for the regeneration and repair of gut epithelial cells (71). As one of the SCFAs produced by the microbiota, butyrate is essential for maintaining gastrointestinal health due to its ability to enhance epithelial barrier integrity and inhibit inflammation (74). *Blautia* is an anaerobic bacterium with the potential to inhibit the colonization of pathogenic bacteria in the intestine and regulate host health (75). The relative abundance of *Blautia* was nearly 20-fold less in the Young group compared to the Cub group, which may affect the gut microbiota homeostasis in young pandas. In support of this conclusion, the relative abundance of pathogenic bacteria, such as *Moraxella* (76) and *Neisseria* (77), was highest in the Young group. Cubs to young giant pandas undergo a dietary transition leading to a fluctuation in the gut microbiota. This fluctuation and rebuilding of the gut microbiota community may have considerable impacts on intestinal homeostasis, resulting in a pathogenic bacterial infection. This may contribute to diarrhea and several gastrointestinal disorders that are often experienced by young captive pandas (68).

### Metabolome and metagenome results indicate inflammation, oxidative stress, and accumulating ARGs in old giant pandas

Metabolome data indicated a distinct metabolite profile in the Old group, with accumulating oxidative and inflammation-related metabolites in the older individuals. Metabolites associated with inflammation, such as kynurenine and leukotriene d4 methyl ester, were significantly higher in the Old group compared to other ages. Kynurenine is a metabolite participating in tryptophan metabolism and mediating various immunomodulatory effects (10, 78). The increased concentration of kynurenine might result from increasing tryptophan degradation with age (79). Tryptophan metabolism causes impaired immune function in aging individuals by decomposition into kynurenine (80). We also found kynurenine content was high in cubs, suggesting potential inflammation in cubs as well as old pandas. Potential inflammation in cubs may be caused by the immaturity of immune function. Leukotriene d4 methyl ester was higher in the Old group and was also significantly positively correlated within the Old group as identified by the WGCNA. Leukotriene d4 methyl ester can cause increased vascular permeability and vasodilatation, playing a vital role in allergic and inflammatory processes (81). The elevation of both metabolites suggests that the old giant pandas were experiencing inflammation. Previous studies based on metabolome of different aged macaques (82) and on transcriptome of different aged giant pandas (5) also suggested geriatric animals had significant inflammation compared to young adults, which is consistent with our results.

We also identified SDMs such as hypoxanthine and L-pyroglutamic acid, they were involved in oxidative stress and were significantly increased abundances in the Old group compared with other age groups. Hypoxanthine showed lowest abundance in the Adult group, while the accumulation of hypoxanthine in the Old group may cause damage by enhancing the production of radicals (83). Hypoxanthine and xanthine are elevated with age in humans and mice and may accumulate reactive oxygen species (84). In addition, L-pyroglutamic acid is the metabolite involved in the glutathione metabolic pathway, and this pathway was enriched in the Old group. L-pyroglutamic acid is an important reducing substance in scavenging oxygen free radicals in animals (85). Congruent with metabolomics results, microbiome functional profiling identified 88 metabolic pathways that were significantly different between the four age groups. Of these pathways, palmitate biosynthesis was significantly enriched in the Old group. The biosynthesis of palmitate can induce the production of reactive oxidative species and plays an important role in the production of reactive oxidative substances (86). Palmitate can trigger oxidative stress and mitochondrial dysfunction (87 - 89). Consequently, our results suggest that the old pandas maybe under oxidative stress.

*Weissella* was significantly different across the four age groups, with the relative abundance increasing from the Cub (0.092% ± 0.002) to Young (1.73% ± 0.046), to Adult (0.56% ± 0.013), and then to the Old group (1.16% ± 0.026). The Young and the Old group had higher abundances of *Weissella* than other age groups probably associated with the dietary transition in the Young group or age-related stresses in the Old group. In addition, the correlation network identified that *Weissella* was the hub bacteria that had the most correlations with SDMs. For example, *Weissella* was positively correlated with Fahfa36:2 abundance and both increased from Cub to Old groups. Fahfa is a newly discovered lipid metabolite, whose synthesis and alteration are involved in the growth and aging process in mice (90). Given the central position of *Weissella* to many age-related SDMs and as an important age-associated microbe, it could be used as a potential age-related biomarker in giant pandas in the future. However, the roles of *Weissella* in aging and how it relates to health in giant pandas remain unclear.

Our study found accumulating ARGs with increasing age in giant pandas. Both the number and the diversity of ARGs increased with age, suggesting a higher risk of bacterial resistance to antibiotics in old pandas. Our results were consistent with previous metagenome studies on giant pandas (23, 91, 92). In particular, we found that the proportion of ARGs belonging to fluoroquinolone, tetracycline, and macrolides antibiotics was high in all giant panda fecal samples. Consequently, the high proportion of ARGs in all samples may hinder the care of captive giant pandas and our findings will provide a foundation for further research.

### Correlation analysis of metabolome and metagenome highlights the involvement of microbiota-bile acid axis in age-related changes

Lipids are important components in animals, and lipids participate in the regulation of many cellular processes (93). Many studies have found that lipid metabolism significantly changes with age in humans and animals. Lipid content is very low in bamboo and although bamboo is the main food source of giant pandas and the species have been extensively studied, the mechanism of lipid metabolism regulation is unclear. Bile acids (BAs) are synthesized by the liver and secreted in the form of primary bile acids or in combination with glycine or taurine (94). BAs enter the digestive tract to regulate the digestion and absorption of lipids and participate in lipid metabolism (95). Ninety-five percent of BAs can be reabsorbed in the jejunum through enterohepatic circulation, and the remaining BAs enter the ileum and colon to be converted into secondary bile acids by bile saline lytic enzyme (BSH) secreted by gut microbes and finally excreted in the form of feces (96). Therefore, the gut microbiota-bile acid axis is involved in, and affects, lipid metabolism and lipid homeostasis. BAs can inhibit the growth of gut microbiota and change its composition, and they can exert an antibacterial effect indirectly through bile acid-related receptors. On the other hand, gut microbiota can change the synthesis and metabolism of BAs by affecting bile acid-related receptors. Our study indicated for the first time that the gut microbiota-bile acid axis plays an important role in age-dependent metabolic regulation in giant pandas. Metabolomic results showed significant differences in the content of BAs across the four age groups, and the SDMs were enriched in primary bile acid synthesis and secondary bile acid synthesis pathways. Mfuzz analysis found BA metabolites belonged to Cluster 3, with the lowest abundance in the Adult, and highest in the Old group. BA metabolites were significantly upregulated SDMs in Old pandas. Meanwhile, the correlation analysis between SDMs and differential gut microbiota showed that BAs were significantly associated with various gut microbiota. For example, Glymodeoxycholic acid, Taurodeoxycholic acid, and Taurodeoxycholic acid were significantly positively correlated with *Lactobacillus* or *Bifidobacterium*, while they were negatively correlated with *Faecalibacterium*. The significant increase in BAs in old giant pandas may be related to the increased occurrence of lipid metabolism disorders in old age. Due to the impairment of lipid metabolism in old pandas, BAs reabsorbed through enterohepatic circulation may be reduced, which resulted in the increase of BAs in the feces. The reduction of BA reabsorption would cause an impact on lipid digestion and absorption and be detrimental to the dissolution of cholesterol. In support of this hypothesis, Huang et al. (5) found that cholesterol levels of old giant pandas were significantly elevated compared to adult giant pandas. The increased excretion of BAs in old pandas also affected the composition of gut microbiota, increasing the relative abundance of *Lactobacillus* and *Bifidobacterium*. Both bacteria can produce BSH (97), and BSH is involved in the dissociation of primary BAs into secondary BAs, including deoxycholic acid (DCA) and lithocholic acid (LCA) (98). This also might contribute to the increased BAs in the feces of old pandas. However, secondary BAs may be involved in the development of a variety of intestinal diseases through various mechanisms. Cao et al. found that deoxycholic acid (DCA)-fed mice had altered gut microbiota composition, associated with impaired intestinal barrier, intestinal inflammation, and adenoma-cancer progression (99). In addition, secondary BAs can activate epidermal growth factor receptor (EGFR) and stimulate NADP(H) oxidase to increase the production of reactive oxygen species (ROS) (100), which can cause DNA damage and inflammation. This was consistent with our results of oxidative stress and inflammation observed in old giant pandas based on the metabolome.

In summary, we characterized metabolite profiles across the giant panda lifespan by combining multi-omics data from UPLC-MS-based metabolomics, 16S rRNA sequencing, and metagenome from giant pandas divided into four age groups. We found that metabolites and the composition/function of gut microbiota changed significantly in response to the transition from a milk-dominant diet in cubs to a bamboo-specific diet in young and adult pandas and that old pandas experienced inflammation, oxidative stress, and accumulating ARGs. Finally, our study highlights that the microbiota-bile acid axis is highly important in age-related changes in giant pandas and provides new insights into the interactions of gut microbiota and metabolites throughout the giant panda’s lifespan.

## Data Availability

The raw sequencing reads from this study have been submitted to the CNGBdb with the project accession CNP0003316.

## SUPPLEMENTAL MATERIAL

Figure S1 msystems.00252-23-s0001.jpg OPLS-DA score plots of fecal metabolites between the different age groups.

Figure S2 msystems.00252-23-s0002.jpg (A) The sobs dilution curve of the sample. (B) The species richness curve of the sample. (C) Boxplot of Ace index between four groups.(D) Boxplot of Shannon index between four groups. (E) Boxplot of Simpson index between four groups. (F) The trend chart of Faith_pd index with age. (G) PLS-DA score plots of gut microbiota between the different age groups.

Figure S3 msystems.00252-23-s0003.jpg (A) LEfSe difference analysis results of KEGG pathway (LDA > 2.0, *P* < 0.05). (B) Circos of GHs distribution in each sample with Top10 relative abundance. (C) Heatmaps of the relative abundance of ARGs with Top20 relative abundance in each sample. (D) CAZymes differ significantly between Cub and Young, Young and Adult, and Adult and Old with *P* < 0.05.

Figure S4 msystems.00252-23-s0004.jpg Differential analysis results of pathway annotated by the HUMAnN2 pipeline using LEfSe (LDA > 2.0, *P* < 0.05).

Table S1 msystems.00252-23-s0005.xlsx The sample information.

Table S2 msystems.00252-23-s0006.xlsx The SDMs between different groups.

Table S3 msystems.00252-23-s0007.xlsx The KEGG enrichment information of SDMs in different clusters.

Table S4 msystems.00252-23-s0008.xlsx The information of significance modules.

Table S5 msystems.00252-23-s0009.xlsx The metagenomics data summary information.

Table S6 msystems.00252-23-s0010.xlsx The correlation results of SDMs and different genera between any two compare groups at four age stages of giant panda.

ASM does not own the copyrights to Supplemental Material that may be linked to, or accessed through, an article. The authors have granted ASM a non-exclusive, world-wide license to publish the Supplemental Material files. Please contact the corresponding author directly for reuse.

## References

[1] Fei Y , Hou R , Spotila JR , Paladino FV , Qi D , Zhang Z . 2016. Metabolic rates of giant pandas inform conservation strategies. Sci Rep 6:27248. doi:10.1038/srep27248 27264109 PMC4893702

[2] Nie Y , Speakman JR , Wu Q , Zhang C , Hu Y , Xia M , Yan L , Hambly C , Wang L , Wei W , Zhang J , Wei F . 2015. Exceptionally low daily energy expenditure in the bamboo-eating giant panda. Science 349:171–174. doi:10.1126/science.aab2413 26160943

[3] He X , Hsu WH , Hou R , Yao Y , Xu Q , Jiang D , Wang L , Wang H . 2020. Comparative genomics reveals bamboo feeding adaptability in the giant panda (Ailuropoda melanoleuca). Zookeys 923:141–156. doi:10.3897/zookeys.923.39665 32292275 PMC7142162

[4] Du L , Liu Q , Shen F , Fan Z , Hou R , Yue B , Zhang X . 2019. Transcriptome analysis reveals immune-related gene expression changes with age in giant panda (Ailuropoda melanoleuca) blood. Aging (Albany NY) 11:249–262. doi:10.18632/aging.101747 30641486 PMC6339791

[5] Huang X , Ouyang Q , Ran M , Zeng B , Deng L , Hu S , Yang M , Li G , Deng T , He M , Li T , Yang H , Zhang G , Zhang H , Zeng C , Wang J . 2020. The immune and metabolic changes with age in giant panda blood by combined transcriptome and DNA methylation analysis. Aging (Albany NY) 12:21777–21797. doi:10.18632/aging.103990 33188156 PMC11623972

[6] Zhang W , Liu W , Hou R , Zhang L , Schmitz-Esser S , Sun H , Xie J , Zhang Y , Wang C , Li L , Yue B , Huang H , Wang H , Shen F , Zhang Z . 2018. Age-associated microbiome shows the giant panda lives on hemicelluloses, not on cellulose. ISME J 12:1319–1328. doi:10.1038/s41396-018-0051-y 29391488 PMC5931968

[7] Zhou Y-Z , Yan M-L , Gao L , Zhang J-Q , Qin X-M , Zhang X , Du G-H . 2017. Metabonomics approach to assessing the metabolism variation and gender gap of Drosophila melanogaster in aging process. Exp Gerontol 98:110–119. doi:10.1016/j.exger.2017.07.020 28811139

[8] Zhang T , Zhang R , Zhang L , Zhang Z , Hou R , Wang H , Loeffler IK , Watson DG , Kennedy MW . 2015. Changes in the milk Metabolome of the giant panda (Ailuropoda melanoleuca) with time after birth--three phases in early Lactation and progressive individual differences. PLoS One 10:e0143417. doi:10.1371/journal.pone.0143417 26630345 PMC4668050

[9] Ball HC , Levari-Shariati S , Cooper LN , Aliani M . 2018. Comparative metabolomics of aging in a long-lived bat: Insights into the physiology of extreme longevity. PLoS One 13:e0196154. doi:10.1371/journal.pone.0196154 29715267 PMC5929510

[10] Hoffman JM , Tran V , Wachtman LM , Green CL , Jones DP , Promislow DEL . 2016. A longitudinal analysis of the effects of age on the blood plasma metabolome in the common marmoset, Callithrix jacchus. Exp Gerontol 76:17–24. doi:10.1016/j.exger.2016.01.007 26805607 PMC4775367

[11] Johnson AA , Stolzing A . 2019. The role of lipid metabolism in aging, LifeSpan regulation, and age-related disease. Aging Cell 18:e13048. doi:10.1111/acel.13048 31560163 PMC6826135

[12] Clement J , Wong M , Poljak A , Sachdev P , Braidy N . 2019. The plasma NAD (+) metabolome is dysregulated in "normal'' aging. Rejuvenation Res 22:121–130. doi:10.1089/rej.2018.2077 30124109 PMC6482912

[13] Visconti A , Le Roy CI , Rosa F , Rossi N , Martin TC , Mohney RP , Li W , de Rinaldis E , Bell JT , Venter JC , Nelson KE , Spector TD , Falchi M . 2019. Interplay between the human gut microbiome and host metabolism. Nat Commun 10:4505. doi:10.1038/s41467-019-12476-z 31582752 PMC6776654

[14] Brandsma E , Kloosterhuis NJ , Koster M , Dekker DC , Gijbels MJJ , van der Velden S , Ríos-Morales M , van Faassen MJR , Loreti MG , de Bruin A , Fu J , Kuipers F , Bakker BM , Westerterp M , de Winther MPJ , Hofker MH , van de Sluis B , Koonen DPY . 2019. A proinflammatory gut microbiota increases systemic inflammation and accelerates atherosclerosis. Circ Res 124:94–100. doi:10.1161/CIRCRESAHA.118.313234 30582442 PMC6325767

[15] Sato Y , Atarashi K , Plichta DR , Arai Y , Sasajima S , Kearney SM , Suda W , Takeshita K , Sasaki T , Okamoto S , Skelly AN , Okamura Y , Vlamakis H , Li Y , Tanoue T , Takei H , Nittono H , Narushima S , Irie J , Itoh H , Moriya K , Sugiura Y , Suematsu M , Moritoki N , Shibata S , Littman DR , Fischbach MA , Uwamino Y , Inoue T , Honda A , Hattori M , Murai T , Xavier RJ , Hirose N , Honda K . 2021. Novel bile acid biosynthetic pathways are enriched in the microbiome of centenarians. Nature 599:458–464. doi:10.1038/s41586-021-03832-5 34325466

[16] Ðanić M , Stanimirov B , Pavlović N , Goločorbin-Kon S , Al-Salami H , Stankov K , Mikov M . 2018. Pharmacological applications of bile acids and their derivatives in the treatment of metabolic syndrome. Front Pharmacol 9:1382. doi:10.3389/fphar.2018.01382 30559664 PMC6287190

[17] Honda A , Miyazaki T , Iwamoto J , Hirayama T , Morishita Y , Monma T , Ueda H , Mizuno S , Sugiyama F , Takahashi S , Ikegami T . 2020. Regulation of bile acid metabolism in mouse models with hydrophobic bile acid composition. J Lipid Res 61:54–69. doi:10.1194/jlr.RA119000395 31645370 PMC6939601

[18] Cao M , Li C , Liu Y , Cai K , Chen L , Yuan C , Zhao Z , Zhang B , Hou R , Zhou X . 2020. Assessing urinary metabolomics in giant pandas using chromatography/mass spectrometry: pregnancy-related changes in the metabolome. Front Endocrinol (Lausanne) 11:215. doi:10.3389/fendo.2020.00215 32373070 PMC7176934

[19] Wang H , Zhong H , Hou R , Ayala J , Liu G , Yuan S , Yan Z , Zhang W , Liu Y , Cai K , Cai Z , Huang H , Zhang Z , Wu D . 2017 A diet diverse in bamboo parts is important for giant panda (Ailuropoda melanoleuca) metabolism and health. Sci Rep 7:3377. doi:10.1038/s41598-017-03216-8 28611401 PMC5469786

[20] Wang L , Huang G , Hou R , Qi D , Wu Q , Nie Y , Zuo Z , Ma R , Zhou W , Ma Y , Hu Y , Yang Z , Yan L , Wei F . 2021. Multi-omics reveals the positive leverage of plant secondary metabolites on the gut microbiota in a non-model mammal. Microbiome 9:192. doi:10.1186/s40168-021-01142-6 34548111 PMC8456708

[21] Zhu C , Laghi L , Zhang Z , He Y , Wu D , Zhang H , Huang Y , Li C , Zou L . 2020. First steps toward the giant panda metabolome database: untargeted metabolomics of feces, urine, serum, and saliva by ^1^H NMR. J Proteome Res 19:1052–1059. doi:10.1021/acs.jproteome.9b00564 31994893

[22] Yang S , Huang Y , Li C , Jin L , Deng W , Zhao S , Wu D , He Y , Li B , Yu Z , Li T , Zhang Z , Pan X , Zhang H , Zou L . 2021. The fecal and serum metabolomics of giant pandas based on untargeted metabolomics. Zoolog Sci 38:179–186. doi:10.2108/zs200106 33812357

[23] Guo M , Chen J , Li Q , Fu Y , Fan G , Ma J , Peng L , Zeng L , Chen J , Wang Y , Lee S-Y . 2018. Dynamics of gut microbiome in giant panda cubs reveal transitional microbes and pathways in early life. Front Microbiol 9:3138. doi:10.3389/fmicb.2018.03138 30619206 PMC6305432

[24] Winston JA , Rivera A , Cai J , Patterson AD , Theriot CM . 2021. Secondary bile acid ursodeoxycholic acid alters weight, the gut microbiota, and the bile acid pool in conventional mice. PLoS One 16:e0246161. doi:10.1371/journal.pone.0246161 33600468 PMC7891722

[25] Domingo-Almenara X , Siuzdak G . 2020. Metabolomics data processing using XCMS. Methods Mol Biol 2104:11–24. doi:10.1007/978-1-0716-0239-3_2 31953810

[26] Kanehisa M . 2017. Enzyme annotation and metabolic reconstruction using KEGG. Methods Mol Biol 1611:135–145. doi:10.1007/978-1-4939-7015-5_11 28451977

[27] Xia J , Wishart DS . 2010. MSEA: a web-based tool to identify biologically meaningful patterns in quantitative metabolomic data. Nucleic Acids Res 38:W71–W77. doi:10.1093/nar/gkq329 20457745 PMC2896187

[28] Kumar L , E Futschik M . 2007. Mfuzz: a software package for soft clustering of microarray data. Bioinformation 2:5–7. doi:10.6026/97320630002005 18084642 PMC2139991

[29] Langfelder P , Horvath S . 2008. WGCNA: An R package for weighted correlation network analysis. BMC Bioinformatics 9:559. doi:10.1186/1471-2105-9-559 19114008 PMC2631488

[30] Caporaso JG , Lauber CL , Walters WA , Berg-Lyons D , Lozupone CA , Turnbaugh PJ , Fierer N , Knight R . 2011. Global patterns of 16S rRNA diversity at a depth of millions of sequences per sample. Proc Natl Acad Sci U S A 108:4516–4522. doi:10.1073/pnas.1000080107 20534432 PMC3063599

[31] Bolyen E , Rideout JR , Dillon MR , Bokulich NA , Abnet CC , Al-Ghalith GA , Alexander H , Alm EJ , Arumugam M , Asnicar F , Bai Y , Bisanz JE , Bittinger K , Brejnrod A , Brislawn CJ , Brown CT , Callahan BJ , Caraballo-Rodríguez AM , Chase J , Cope EK , Da Silva R , Diener C , Dorrestein PC , Douglas GM , Durall DM , Duvallet C , Edwardson CF , Ernst M , Estaki M , Fouquier J , Gauglitz JM , Gibbons SM , Gibson DL , Gonzalez A , Gorlick K , Guo J , Hillmann B , Holmes S , Holste H , Huttenhower C , Huttley GA , Janssen S , Jarmusch AK , Jiang L , Kaehler BD , Kang KB , Keefe CR , Keim P , Kelley ST , Knights D , Koester I , Kosciolek T , Kreps J , Langille MGI , Lee J , Ley R , Liu Y-X , Loftfield E , Lozupone C , Maher M , Marotz C , Martin BD , McDonald D , McIver LJ , Melnik AV , Metcalf JL , Morgan SC , Morton JT , Naimey AT , Navas-Molina JA , Nothias LF , Orchanian SB , Pearson T , Peoples SL , Petras D , Preuss ML , Pruesse E , Rasmussen LB , Rivers A , Robeson MS , Rosenthal P , Segata N , Shaffer M , Shiffer A , Sinha R , Song SJ , Spear JR , Swafford AD , Thompson LR , Torres PJ , Trinh P , Tripathi A , Turnbaugh PJ , Ul-Hasan S , van der Hooft JJJ , Vargas F , Vázquez-Baeza Y , Vogtmann E , von Hippel M , Walters W , Wan Y , Wang M , Warren J , Weber KC , Williamson CHD , Willis AD , Xu ZZ , Zaneveld JR , Zhang Y , Zhu Q , Knight R , Caporaso JG . 2019. Reproducible, interactive, scalable and extensible microbiome data science using QIIME 2. Nat Biotechnol 37:852–857. doi:10.1038/s41587-019-0209-9 31341288 PMC7015180

[32] Callahan BJ , McMurdie PJ , Rosen MJ , Han AW , Johnson AJA , Holmes SP . 2016. DADA2: high-resolution sample inference from illumina amplicon data. Nat Methods 13:581–583. doi:10.1038/nmeth.3869 27214047 PMC4927377

[33] Oksanen J , Blanchet FG , Kindt R , Legendre P , Minchin P , O’Hara RB . 2013. Vegan: community ecology package. R Foundation for Statistical Computing, Vienna.

[34] Segata N , Izard J , Waldron L , Gevers D , Miropolsky L , Garrett WS , Huttenhower C . 2011. Metagenomic biomarker discovery and explanation. Genome Biol 12:R60. doi:10.1186/gb-2011-12-6-r60 21702898 PMC3218848

[35] Oksanen J , Blanchet FG , Kindt R , Legendre P , Wagner H . 2013. Vegan: community ecology R package, v2, p 0–10

[36] Wickham H . 2009. Ggplot2: elegant graphics for data analysis. Biometrics, New York, NY. doi:10.1007/978-0-387-98141-3

[37] Gu Z , Eils R , Schlesner M . 2016. Complex heatmaps reveal patterns and correlations in multidimensional genomic data. Bioinformatics 32:2847–2849. doi:10.1093/bioinformatics/btw313 27207943

[38] Yuan Y , Chen Y , Yao F , Zeng M , Xie Q , Shafiq M , Noman SM , Jiao X . 2021. Microbiomes and resistomes in biopsy tissue and intestinal lavage fluid of colorectal cancer. Front Cell Dev Biol 9: 736994. doi:10.3389/fcell.2021.736994 34604238 PMC8484797

[39] Langmead B , Salzberg SL . 2012. Fast gapped-read alignment with Bowtie 2. Nat Methods 9:357–359. doi:10.1038/nmeth.1923 22388286 PMC3322381

[40] Andrews S . 2013. Babraham bioinformatics -fastQC A quality control tool for high throughput sequence data

[41] Wood DE , Salzberg SL . 2014. Kraken:ultrafast metagenomic sequence classification using exact alignments. Genome Biol 15:R46. doi:10.1186/gb-2014-15-3-r46 24580807 PMC4053813

[42] Li D , Liu C-M , Luo R , Sadakane K , Lam T-W . 2015. MEGAHIT: an ultra-fast single-node solution for large and complex metagenomics assembly via succinct de Bruijn graph. Bioinformatics 31:1674–1676. doi:10.1093/bioinformatics/btv033 25609793

[43] Hyatt D , Chen G-L , Locascio PF , Land ML , Larimer FW , Hauser LJ . 2010. Prodigal:prokaryotic gene recognition and translation initiation site identification. BMC Bioinformatics 11:119. doi:10.1186/1471-2105-11-119 20211023 PMC2848648

[44] Li W , Godzik A . 2006. Cd-hit: a fast program for clustering and comparing large sets of protein or nucleotide sequences. Bioinformatics 22:1658–1659. doi:10.1093/bioinformatics/btl158 16731699

[45] Ge H , Sun L , Yu J . 2017. Fast batch searching for protein Homology based on compression and clustering. BMC Bioinformatics 18:508. doi:10.1186/s12859-017-1938-8 29162030 PMC5697088

[46] Franzosa EA , McIver LJ , Rahnavard G , Thompson LR , Schirmer M , Weingart G , Lipson KS , Knight R , Caporaso JG , Segata N , Huttenhower C . 2018. Species-level functional profiling of metagenomes and metatranscriptomes. Nat Methods 15:962–968. doi:10.1038/s41592-018-0176-y 30377376 PMC6235447

[47] Buchfink B , Xie C , Huson DH . 2015. Fast and sensitive protein alignment using DIAMOND. Nat Methods 12:59–60. doi:10.1038/nmeth.3176 25402007

[48] Suzek BE , Wang Y , Huang H , McGarvey PB , Wu CH , UniProt Consortium . 2015. UniRef clusters: a comprehensive and scalable alternative for improving sequence similarity searches. Bioinformatics 31:926–932. doi:10.1093/bioinformatics/btu739 25398609 PMC4375400

[49] Beghini F , McIver LJ , Blanco-Míguez A , Dubois L , Asnicar F , Maharjan S , Mailyan A , Manghi P , Scholz M , Thomas AM , Valles-Colomer M , Weingart G , Zhang Y , Zolfo M , Huttenhower C , Franzosa EA , Segata N . 2021. Integrating taxonomic, functional, and strain-level profiling of diverse microbial communities with bioBakery 3. Elife 10:e65088. doi:10.7554/eLife.65088 33944776 PMC8096432

[50] Kruskal WH , Wallis WA . 1952. Use of ranks in one-criterion variance analysis. J Am Stat Assoc 47:583–621. doi:10.1080/01621459.1952.10483441

[51] Benjamini Y , Hochberg Y . 1995. Controlling the false discovery rate: a practical and powerful approach to multiple testing. J R Stat Soc Series B 57:289–300. doi:10.1111/j.2517-6161.1995.tb02031.x

[52] Parks DH , Tyson GW , Hugenholtz P , Beiko RG . 2014. STAMP: statistical analysis of taxonomic and functional profiles. Bioinformatics 30:3123–3124. doi:10.1093/bioinformatics/btu494 25061070 PMC4609014

[53] Wang S , Tang F , Yue Y , Yao X , Wei Q , Yu J . 2013. Simultaneous determination of 12 coumarins in bamboo leaves by HPLC. J AOAC Int 96:942–946. doi:10.5740/jaoacint.12-441 24282929

[54] Fei Y , Hou R , Spotila JR , Paladino FV , Qi D , Zhang Z . 2016. Metabolic rates of giant pandas inform conservation strategies. Sci Rep 6:27248. doi:10.1038/srep27248 27264109 PMC4893702

[55] Nie Y , Speakman JR , Wu Q , Zhang C , Hu Y , Xia M , Yan L , Hambly C , Wang L , Wei W , Zhang J , Wei F . 2015. ANIMAL PHYSIOLOGY. Exceptionally low daily energy expenditure in the bamboo-eating giant panda. Science 349:171–174. doi:10.1126/science.aab2413 26160943

[56] Cao M , Li C , Liu Y , Cai K , Chen L , Yuan C , Zhao Z , Zhang B , Hou R , Zhou X . 2020. Assessing urinary metabolomics in giant pandas using chromatography/mass spectrometry: pregnancy-related changes in the metabolome. Front Endocrinol (Lausanne) 11:215. doi:10.3389/fendo.2020.00215 32373070 PMC7176934

[57] Brial F , Chilloux J , Nielsen T , Vieira-Silva S , Falony G , Andrikopoulos P , Olanipekun M , Hoyles L , Djouadi F , Neves AL , Rodriguez-Martinez A , Mouawad GI , Pons N , Forslund S , Le-Chatelier E , Le Lay A , Nicholson J , Hansen T , Hyötyläinen T , Clément K , Oresic M , Bork P , Ehrlich SD , Raes J , Pedersen OB , Gauguier D , Dumas M-E . 2021. Human and preclinical studies of the host-gut microbiome co-metabolite hippurate as a marker and mediator of metabolic health. Gut 70:2105–2114. doi:10.1136/gutjnl-2020-323314 33975870 PMC8515120

[58] López-López A , Castellote-Bargalló AI , Campoy-Folgoso C , Rivero-Urgël M , Tormo-Carnicé R , Infante-Pina D , López-Sabater MC . 2001. The influence of dietary palmitic acid triacylglyceride position on the fatty acid, calcium and magnesium contents of at term newborn faeces. Early Hum Dev 65:S83–S94. doi:10.1016/s0378-3782(01)00210-9 11755039

[59] Lu B , Wu X , Tie X , Zhang Y , Zhang Y . 2005. Toxicology and safety of anti-oxidant of bamboo leaves. Part 1: acute and subchronic toxicity studies on anti-oxidant of bamboo leaves. Food Chem Toxicol 43:783–792. doi:10.1016/j.fct.2005.01.019 15778019

[60] Wang L , Yuan S , Nie Y , Zhao J , Cao X , Dai Y , Zhang Z , Wei F . 2020. Dietary flavonoids and the altitudinal preference of wild giant pandas in foping national nature reserve, China. Global Ecology and Conservation 22:e00981. doi:10.1016/j.gecco.2020.e00981

[61] Bibbò S , Ianiro G , Giorgio V , Scaldaferri F , Masucci L , Gasbarrini A , Cammarota G . 2016. The role of diet on gut microbiota composition. Eur Rev Med Pharmacol Sci 20:4742–4749. 27906427

[62] Zhang W , Liu W , Hou R , Zhang L , Schmitz-Esser S , Sun H , Xie J , Zhang Y , Wang C , Li L , Yue B , Huang H , Wang H , Shen F , Zhang Z . 2018. Age-associated microbiome shows the giant panda lives on hemicelluloses, not on cellulose. ISME J 12:1319–1328. doi:10.1038/s41396-018-0051-y 29391488 PMC5931968

[63] Guo W , Chen Y , Wang C , Ning R , Zeng B , Tang J , Li C , Zhang M , Li Y , Ni Q , Ni X , Zhang H , Li D , Zhao J , Li Y . 2020. The Carnivorous digestive system and bamboo diet of giant Pandas may shape their low gut bacterial diversity. Conserv Physiol 8:coz104. doi:10.1093/conphys/coz104 32190328 PMC7066643

[64] Jin L , Huang Y , Yang S , Wu D , Li C , Deng W , Zhao K , He Y , Li B , Zhang G , Xiong Y , Wei R , Li G , Wu H , Zhang H , Zou L . 2021. Diet, habitat environment and lifestyle conversion affect the gut microbiomes of giant pandas. Sci Total Environ 770: S0048-9697(21)00383-1. doi:10.1016/j.scitotenv.2021.145316 33517011

[65] Williams CL , Dill-McFarland KA , Vandewege MW , Sparks DL , Willard ST , Kouba AJ , Suen G , Brown AE . 2016. Dietary shifts may trigger dysbiosis and mucous stools in giant pandas (Ailuropoda melanoleuca). Front Microbiol 7:661. doi:10.3389/fmicb.2016.00661 27199976 PMC4858621

[66] Stewart RD , Auffret MD , Warr A , Wiser AH , Press MO , Langford KW , Liachko I , Snelling TJ , Dewhurst RJ , Walker AW , Roehe R , Watson M . 2018. Assembly of 913 microbial genomes from metagenomic sequencing of the cow rumen. Nat Commun 9:870. doi:10.1038/s41467-018-03317-6 29491419 PMC5830445

[67] Niu Q , Li P , Hao S , Zhang Y , Kim SW , Li H , Ma X , Gao S , He L , Wu W , Huang X , Hua J , Zhou B , Huang R . 2015. Dynamic distribution of the gut microbiota and the relationship with apparent crude fiber digestibility and growth stages in pigs. Sci Rep 5:9938. doi:10.1038/srep09938 25898122 PMC4404679

[68] Zhan M , Wang L , Xie C , Fu X , Zhang S , Wang A , Zhou Y , Xu C , Zhang H . 2020. Succession of gut microbial structure in twin giant pandas during the dietary change stage and its role in polysaccharide metabolism. Front Microbiol 11:551038. doi:10.3389/fmicb.2020.551038 33072012 PMC7537565

[69] Li R , Fan W , Tian G , Zhu H , He L , Cai J , Huang Q , Cai Q , Li B , Bai Y , Zhang Z , Zhang Y , Wang W , Li J , Wei F , Li H , Jian M , Li J , Zhang Z , Nielsen R , Li D , Gu W , Yang Z , Xuan Z , Ryder OA , Leung F-C , Zhou Y , Cao J , Sun X , Fu Y , Fang X , Guo X , Wang B , Hou R , Shen F , Mu B , Ni P , Lin R , Qian W , Wang G , Yu C , Nie W , Wang J , Wu Z , Liang H , Min J , Wu Q , Cheng S , Ruan J , Wang M , Shi Z , Wen M , Liu B , Ren X , Zheng H , Dong D , Cook K , Shan G , Zhang H , Kosiol C , Xie X , Lu Z , Zheng H , Li Y , Steiner CC , Lam T-Y , Lin S , Zhang Q , Li G , Tian J , Gong T , Liu H , Zhang D , Fang L , Ye C , Zhang J , Hu W , Xu A , Ren Y , Zhang G , Bruford MW , Li Q , Ma L , Guo Y , An N , Hu Y , Zheng Y , Shi Y , Li Z , Liu Q , Chen Y , Zhao J , Qu N , Zhao S , Tian F , Wang X , Wang H , Xu L , Liu X , Vinar T , Wang Y , Lam T-W , Yiu S-M , Liu S , Zhang H , Li D , Huang Y , Wang X , Yang G , Jiang Z , Wang J , Qin N , Li L , Li J , Bolund L , Kristiansen K , Wong G-S , Olson M , Zhang X , Li S , Yang H , Wang J , Wang J . 2010. The sequence and de novo assembly of the giant panda genome. Nature 463:311–317. doi:10.1038/nature08696 20010809 PMC3951497

[70] Lombard V , Golaconda Ramulu H , Drula E , Coutinho PM , Henrissat B . 2014. The carbohydrate-active enzymes database (CAZy) in 2013. Nucleic Acids Res 42:D490–D495. doi:10.1093/nar/gkt1178 24270786 PMC3965031

[71] Lopez-Siles M , Duncan SH , Garcia-Gil LJ , Martinez-Medina M . 2017. Faecalibacterium prausnitzii: from microbiology to diagnostics and prognostics. ISME J 11:841–852. doi:10.1038/ismej.2016.176 28045459 PMC5364359

[72] Zhang R , Zhang J , Dang W , Irwin DM , Wang Z , Zhang S . 2020. Unveiling the biogeography and potential functions of the intestinal digesta- and mucosa-associated microbiome of donkeys. Front Microbiol 11:596882. doi:10.3389/fmicb.2020.596882 33424800 PMC7793809

[73] Liu X , Mao B , Gu J , Wu J , Cui S , Wang G , Zhao J , Zhang H , Chen W . 2021. Blautia-a new functional genus with potential probiotic properties? Gut Microbes 13:1–21. doi:10.1080/19490976.2021.1875796 PMC7872077 33525961

[74] Hamer HM , Jonkers D , Venema K , Vanhoutvin S , Troost FJ , Brummer R-J . 2008. Review article: the role of butyrate on colonic function. Aliment Pharmacol Ther 27:104–119. doi:10.1111/j.1365-2036.2007.03562.x 17973645

[75] Benítez-Páez A , Gómez Del Pugar EM , López-Almela I , Moya-Pérez Á , Codoñer-Franch P , Sanz Y . 2020. Depletion of blautia species in the microbiota of obese children relates to intestinal inflammation and metabolic phenotype worsening. mSystems 5:e00857-19. doi:10.1128/mSystems.00857-19 32209719 PMC7093825

[76] Ma R , Hou R , Guo J-L , Zhang X-Y , Cao S-J , Huang X-B , Wu R , Wen Y-P , Zhao Q , Du S-Y , Lin J-C , Bai Y , Yan Q-G , Qi D-W . 2022. The plaque microbiota community of giant panda (Ailuropoda melanoleuca) cubs with dental caries. Front Cell Infect Microbiol 12:866410. doi:10.3389/fcimb.2022.866410 35573790 PMC9097603

[77] Zhang L , Li C , Zhai Y , Feng L , Bai K , Zhang Z , Huang Y , Li T , Li D , Li H , Cui P , Chen D , Wang H , Yang X . 2020. Analysis of the vaginal microbiome of giant pandas using metagenomics sequencing. Microbiologyopen 9:e1131. doi:10.1002/mbo3.1131 33205903 PMC7755806

[78] Keszthelyi D , Troost FJ , Masclee AAM . 2009. Understanding the role of tryptophan and serotonin metabolism in gastrointestinal function. Neurogastroenterol Motil 21:1239–1249. doi:10.1111/j.1365-2982.2009.01370.x 19650771

[79] van der Goot AT , Nollen EAA . 2013. Tryptophan metabolism: entering the field of aging and age-related pathologies. Trends Mol Med 19:336–344. doi:10.1016/j.molmed.2013.02.007 23562344

[80] Rivero-Segura NA , Bello-Chavolla OY , Barrera-Vázquez OS , Gutierrez-Robledo LM , Gomez-Verjan JC . 2020. Promising biomarkers of human aging: in search of a multi-omics panel to understand the aging process from a multidimensional perspective. Ageing Res Rev 64:101164. doi:10.1016/j.arr.2020.101164 32977058

[81] Wei J , Chen S , Guo W , Feng B , Yang S , Huang C , Chu J . 2018. Leukotriene D4 induces cellular senescence in osteoblasts. Int Immunopharmacol 58:154–159. doi:10.1016/j.intimp.2017.12.027 29587204

[82] Pallikkuth S , Mendez R , Russell K , Sirupangi T , Kvistad D , Pahwa R , Villinger F , Banerjee S , Pahwa S . 2021. Age associated microbiome and microbial metabolites modulation and its association with systemic inflammation in a rhesus macaque model. Front Immunol 12:748397. doi:10.3389/fimmu.2021.748397 34737748 PMC8560971

[83] Willems L , Garnham B , Headrick JP . 2003. Aging-related changes in myocardial purine metabolism and ischemic tolerance. Exp Gerontol 38:1169–1177. doi:10.1016/j.exger.2003.08.003 14580870

[84] Son N , Hur HJ , Sung MJ , Kim M-S , Hwang J-T , Park JH , Yang HJ , Kwon DY , Yoon SH , Chung HY , Kim H-J . 2012. Liquid chromatography-mass spectrometry-based metabolomic analysis of livers from aged rats. J Proteome Res 11:2551–2558. doi:10.1021/pr201263q 22380686

[85] Traverso N , Ricciarelli R , Nitti M , Marengo B , Furfaro AL , Pronzato MA , Marinari UM , Domenicotti C . 2013. Role of glutathione in cancer progression and chemoresistance. Oxid Med Cell Longev 2013:972913. doi:10.1155/2013/972913 23766865 PMC3673338

[86] Li H , Xiao Y , Tang L , Zhong F , Huang G , Xu J-M , Xu A-M , Dai R-P , Zhou Z-G . 2018. Adipocyte fatty acid-binding protein promotes palmitate-induced mitochondrial dysfunction and apoptosis in macrophages. Front Immunol 9:81. doi:10.3389/fimmu.2018.00081 29441065 PMC5797554

[87] de Pablo MA , Susin SA , Jacotot E , Larochette N , Costantini P , Ravagnan L , Zamzami N , Kroemer G . 1999. Palmitate induces apoptosis via a direct effect on mitochondria. Apoptosis 4:81–87. doi:10.1023/a:1009694124241 14634285

[88] Lambertucci RH , Hirabara SM , Silveira LDR , Levada-Pires AC , Curi R , Pithon-Curi TC . 2008. Palmitate increases superoxide production through mitochondrial electron transport chain and NADPH oxidase activity in skeletal muscle cells. J Cell Physiol 216:796–804. doi:10.1002/jcp.21463 18446788

[89] Yuzefovych L , Wilson G , Rachek L . 2010. Different effects of oleate vs. palmitate on mitochondrial function, apoptosis, and insulin signaling in L6 skeletal muscle cells: role of oxidative stress. Am J Physiol Endocrinol Metab 299:E1096–E1105. doi:10.1152/ajpendo.00238.2010 20876761 PMC3006254

[90] Zhu Q-F , Yan J-W , Ni J , Feng Y-Q . 2020. FAHFA footprint in the visceral fat of mice across their lifespan. Biochim Biophys Acta Mol Cell Biol Lipids 1865:158639. doi:10.1016/j.bbalip.2020.158639 31988049

[91] Hu T , Dai Q , Chen H , Zhang Z , Dai Q , Gu X , Yang X , Yang Z , Zhu L . 2021. Geographic pattern of antibiotic resistance genes in the metagenomes of the giant panda. Microb Biotechnol 14:186–197. doi:10.1111/1751-7915.13655 32812361 PMC7888472

[92] Mustafa GR , Li C , Zhao S , Jin L , He X , Shabbir MZ , He Y , Li T , Deng W , Xu L , Xiong Y , Zhang G , Zhang H , Huang Y , Zou L . 2021. Metagenomic analysis revealed a wide distribution of antibiotic resistance genes and biosynthesis of antibiotics in the gut of giant pandas. BMC Microbiol 21: 15. doi:10.1186/s12866-020-02078-x 33413128 PMC7792088

[93] Huang C , Freter C . 2015. Lipid metabolism, apoptosis and cancer therapy. Int J Mol Sci 16:924–949. doi:10.3390/ijms16010924 25561239 PMC4307283

[94] Di Ciaula A , Garruti G , Lunardi Baccetto R , Molina-Molina E , Bonfrate L , Wang D-H , Portincasa P . 2017. Bile acid physiology. Ann Hepatol 16:s4–s14. doi:10.5604/01.3001.0010.5493 29080336

[95] Chiang JYL . 2013. Bile acid metabolism and signaling. Compr Physiol 3:1191–1212. doi:10.1002/cphy.c120023 23897684 PMC4422175

[96] Hofmann AF . 2009. The enterohepatic circulation of bile acids in mammals: form and functions. Front Biosci (Landmark Ed) 14:2584–2598. doi:10.2741/3399 19273221

[97] Wahlström A , Sayin SI , Marschall H-U , Bäckhed F . 2016. Intestinal crosstalk between bile acids and microbiota and its impact on host metabolism. Cell Metab 24:41–50. doi:10.1016/j.cmet.2016.05.005 27320064

[98] Winston JA , Theriot CM . 2020. Diversification of host bile acids by members of the gut microbiota. Gut Microbes 11:158–171. doi:10.1080/19490976.2019.1674124 31595814 PMC7053883

[99] Cao H , Xu M , Dong W , Deng B , Wang S , Zhang Y , Wang S , Luo S , Wang W , Qi Y , Gao J , Cao X , Yan F , Wang B . 2017. Secondary bile acid-induced dysbiosis promotes intestinal carcinogenesis. Int J Cancer 140:2545–2556. doi:10.1002/ijc.30643 28187526

[100] Ignacio Barrasa J , Olmo N , Pérez-Ramos P , Santiago-Gómez A , Lecona E , Turnay J , Antonia Lizarbe M . 2011. Deoxycholic and chenodeoxycholic bile acids induce apoptosis via oxidative stress in human colon adenocarcinoma cells. Apoptosis 16:1054–1067. doi:10.1007/s10495-011-0633-x 21789651

