# Supplementary figures and images for: Age-related alterations in metabolome and microbiome provide insights in dietary transition in giant pandas
Source: mSystems. 2023 Jun 5;8(3):e00252-23. doi: 10.1128/msystems.00252-23 (PMC10308887; doi:10.1128/msystems.00252-23)

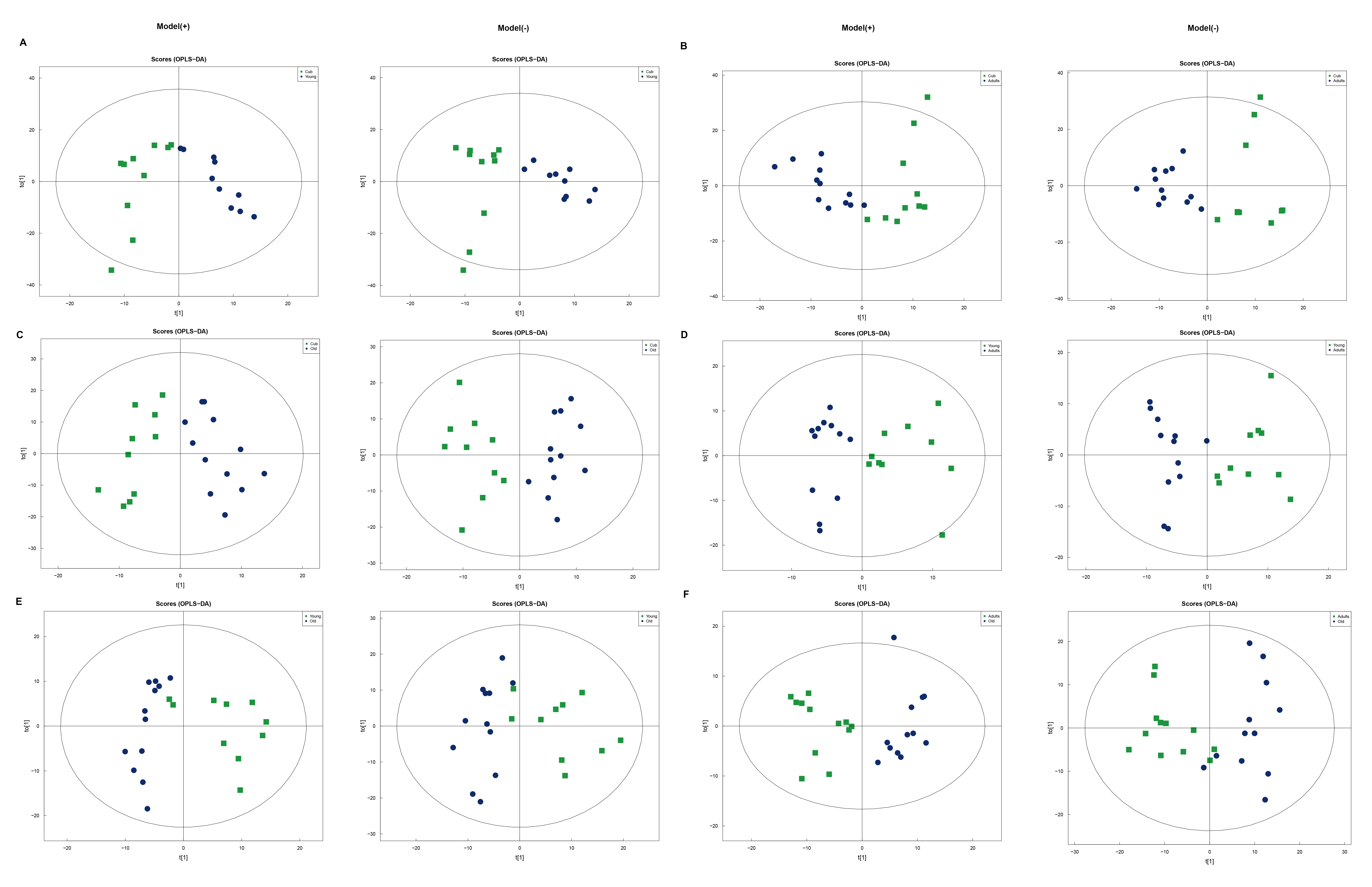

Supplement: Figure S1 — OPLS-DA score plots of fecal metabolites between the different age groups. [file msystems.00252-23-s0001.jpg]

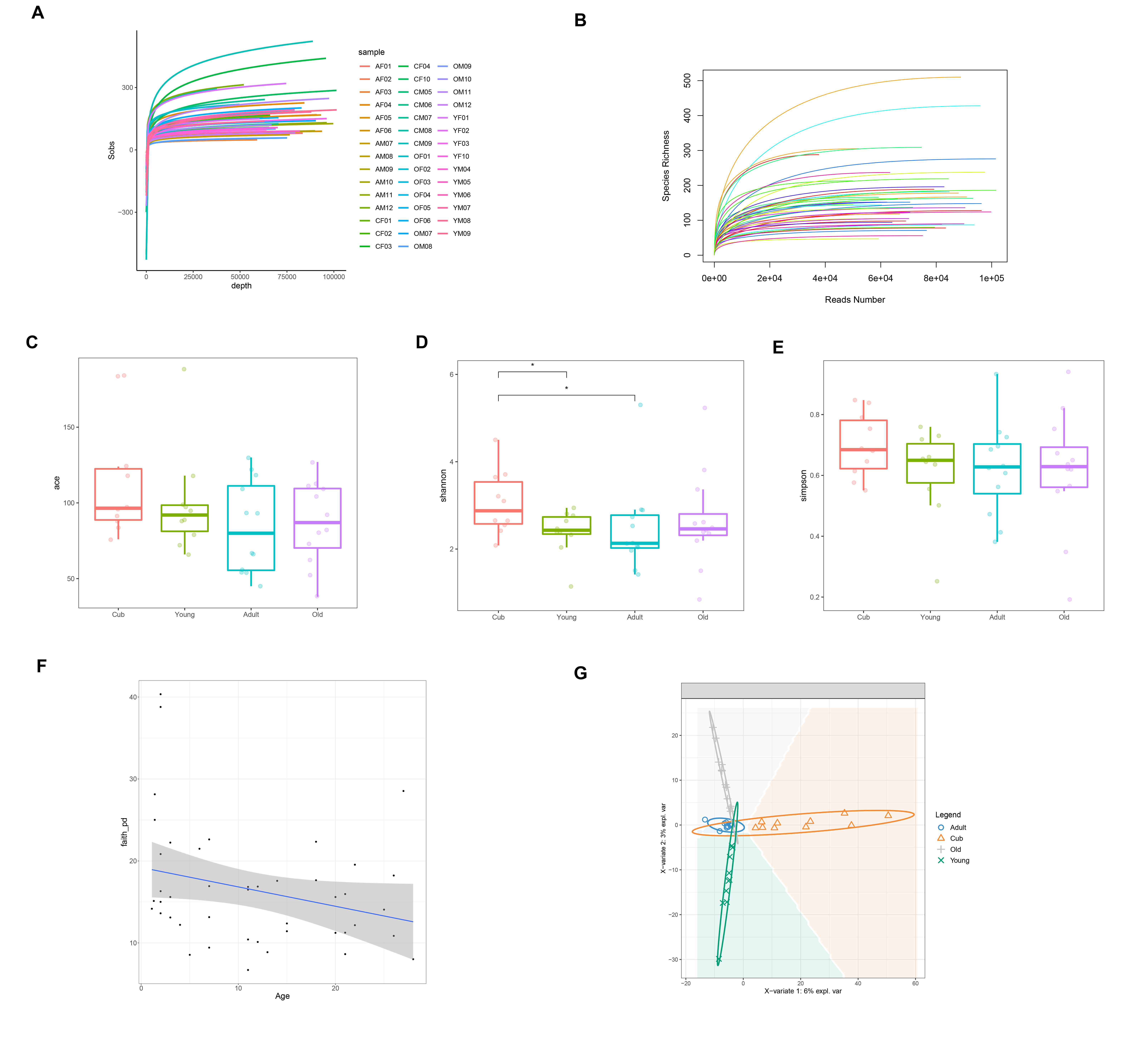

Supplement: Figure S2 — (A) The sobs dilution curve of the sample. (B) The species richness curve of the sample. (C) Boxplot of Ace index between four groups.(D) Boxplot of Shannon index between four groups. (E) Boxplot of Simpson index between four groups. (F) The trend chart of Faith_pd index with age. (G) PLS-DA score plots of gut microbiota between the different age groups. [file msystems.00252-23-s0002.jpg]

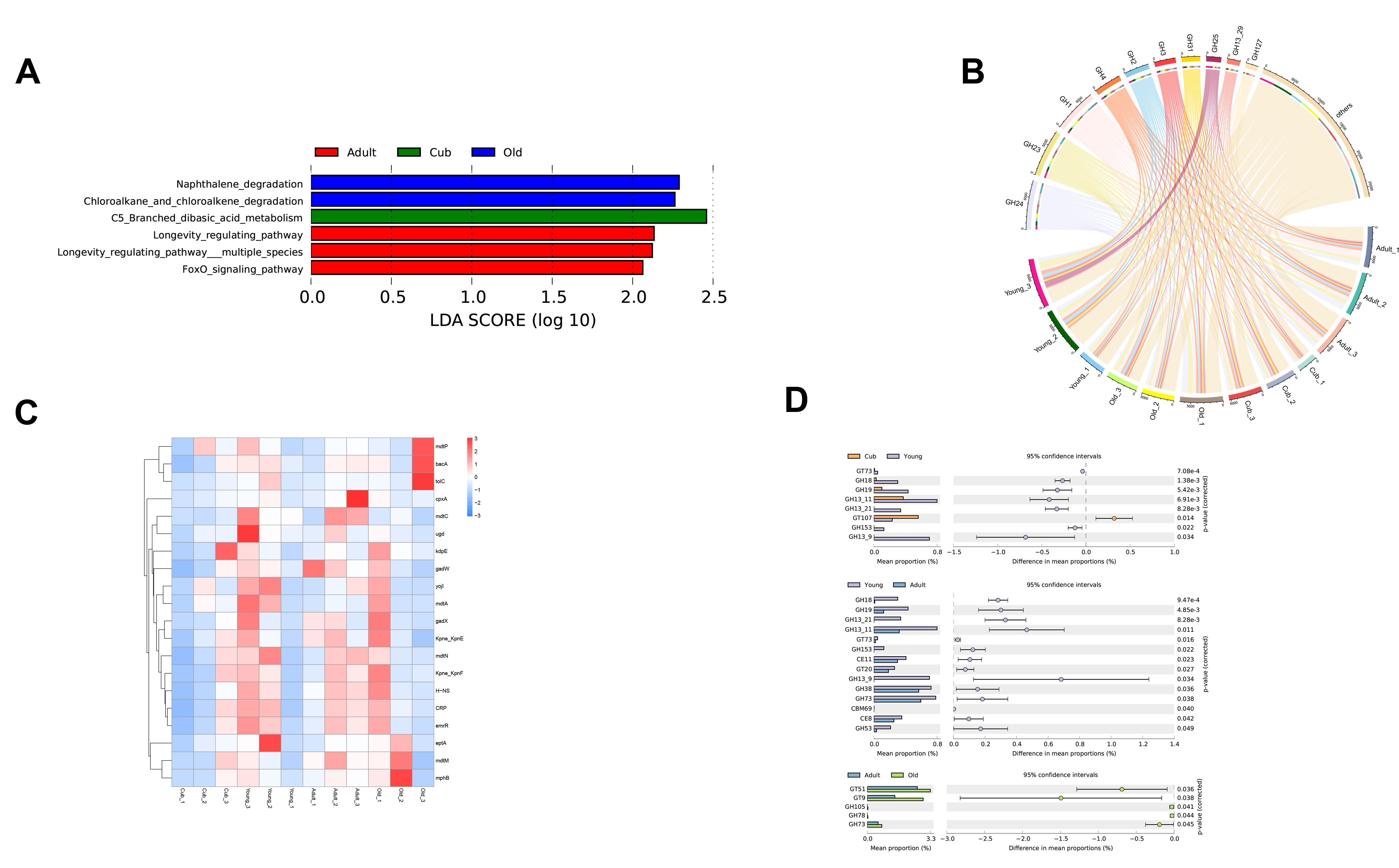

Supplement: Figure S3 — (A) LEfSe difference analysis results of KEGG pathway (LDA > 2.0, P < 0.05). (B) Circos of GHs distribution in each sample with Top10 relative abundance. (C) Heatmaps of the relative abundance of ARGs with Top20 relative abundance in each sample. (D) CAZymes differ significantly between Cub and Young, Young and Adult, and Adult and Old with P < 0.05. [file msystems.00252-23-s0003.jpg]

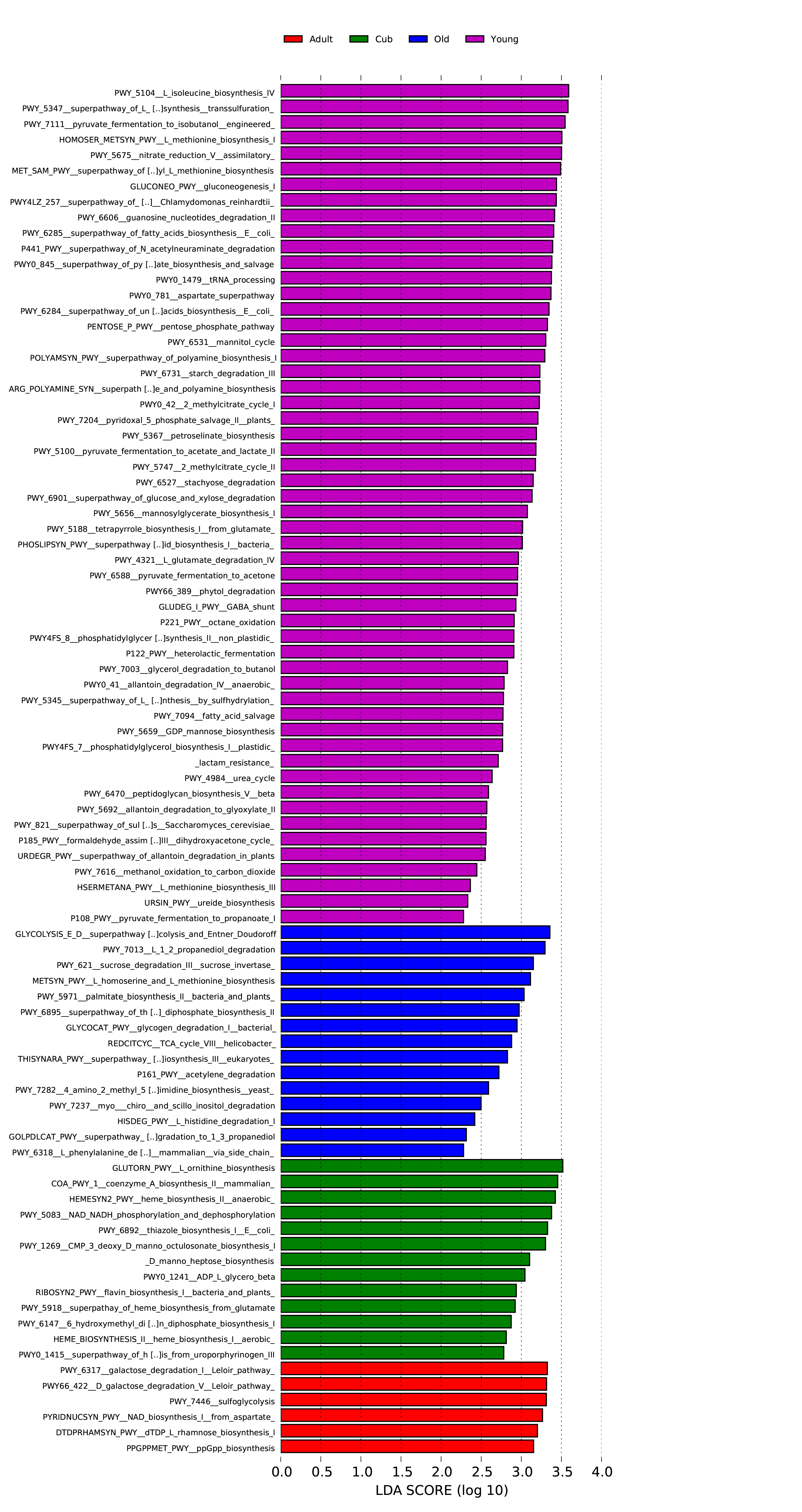

Supplement: Figure S4 — Differential analysis results of pathway annotated by the HUMAnN2 pipeline using LEfSe (LDA > 2.0, P < 0.05). [file msystems.00252-23-s0004.jpg]
